# C3 and CD47 mediate sensory-motor circuit refinement during spinal cord development

**DOI:** 10.1084/jem.20251329

**Published:** 2026-09-16

**Authors:** Danny Florez-Paz, George Z. Mentis

**Affiliations:** 1 Center for Motor Neuron Biology and Disease, Columbia University, New York, NY, USA; 2 Department of Neurology, Columbia University, New York, NY, USA; 3 Department of Pathology and Cell Biology, Columbia University, New York, NY, USA

## Abstract

Overground movement in mammals requires the assembly and refinement of sensory-motor circuits to ensure proper motor control. Although, supernumerary synapses are formed and subsequently pruned in the brain, whether this occurs within spinal sensory-motor circuits remains unclear. Moreover, it is unknown what molecules are involved. Here, we demonstrate the presence of proprioceptive supernumerary synapses forming inappropriate contacts with motor neurons, resulting in miswired immature circuits. Using mouse genetics, neuronal circuit mapping, electrophysiology, and behavioral studies, we demonstrate that the inappropriate synapses are functional, leading to impaired behaviors. We further identify two complementary mechanisms responsible for their elimination: first, C3 through the classical complement pathway and second, CD47 that operates independently of classical complement signaling. This finding underlies an unexpected function for CD47 within the spinal cord, in contrast to its function in the brain. Thus, during early development, the course of elimination of inappropriately generated synapses utilizes a dual fail–safe system to ensure emergence of mature spinal motor circuits.

## Teaser

Florez-Paz and Mentis demonstrate the presence of supernumerary synapses during development, resulting in miswired immature spinal circuits. Two molecularly distinct mechanisms are responsible for their refinement: first, C3, a contributing factor through classical complement activation, and second, a surprising CD47-dependent mechanism, which contributes to synaptic elimination, in contrast to its protective effect in the brain.

## Introduction

Smooth and flawless overground movement in mammals requires a timely and carefully orchestrated assembly and refinement of spinal sensory-motor circuits. Sensory neurons, spinal interneurons, and motor neurons are key players that form intricate neuronal circuits during embryonic development (Frank 1993). These circuits are fine-tuned during early postnatal development, at a time when the animal is moving independently (Robinson et al., 2000; Inacio et al., 2016; Blankenship and Feller 2010). Early formation of neuronal circuits is driven by specific molecular mechanisms (Jessell 2000; Lee and Pfaff 2001; Arber 2012). In sensory-motor circuits embedded within the spinal cord, information from the periphery, originating from proprioceptive and tactile sensory neurons, is essential to the generation of smooth movement and key to counteracting perturbations during locomotion (Mayer et al., 2018; Akay et al., 2014; Takeoka and Arber 2019; Gradwell et al., 2024). Proprioceptors are a select set of sensory neurons that convey information about muscle stretch and tension, two metrics that enable the CNS to estimate limb kinetics and position (de Nooij and Zampieri 2023; Proske and Gandevia 2012; Oliver et al., 2021). Aside from supraspinal targets, proprioceptive neurons also feed into spinal reflex circuits through contacts with several classes of spinal interneurons (Binder 1980; Bikoff et al., 2016; Mentis et al., 2010; Siembab et al., 2010) and direct monosynaptic contacts with motor neurons (Eccles et al. 1957; Mears and Frank 1997).

In mature neuronal circuits, each motor neuron receives proprioceptive inputs that originate from the same (homonymous) muscle innervated by the motor neuron, as well as from proprioceptive fibers arising from synergistic muscles (Mears and Frank 1997; Mendelsohn et al., 2015). While adult motor neurons do not receive proprioceptive inputs from antagonistic muscles, during embryonic and postnatal development this is not the case. Previous studies reported retraction of proprioceptive synapses from the ventral horn during postnatal development (Chen and Frank 1999; Seebach and Ziskind-Conhaim 1994; Gibson and Clowry 1999) as well as the dorsal and intermediate regions of the cervical spinal cord (Chakrabarty and Martin 2011). Whether these supernumerary sensory synapses that are eliminated during spinal cord development represent inappropriately formed synapses or include appropriately formed ones, which are pruned during development, is not well understood. Furthermore, it is unknown whether supernumerary synapses could form inappropriate contacts, leading to miswired neuronal circuits. Could inappropriate synapses be eliminated as part of synaptic refinement and the emergence of mature sensory-motor circuits? Importantly, the molecular mechanisms responsible for the refinement of spinal sensory-motor circuits and synaptic elimination remain poorly understood.

C1q, the initiating protein of the classical complement cascade, has been reported to tag synapses in several neuronal circuits, including the visual system and cortical circuits (Fonseca et al., 2017; Stephan et al., 2013; Stevens et al., 2007). Furthermore, we have previously reported that C1q is implicated in synaptic dysfunction and subsequent elimination during normal development and in the neurodegenerative disease spinal muscular atrophy (Vukojicic et al., 2019). However, whether the classical complement is involved would require investigation of at least one downstream protein from C1q, and for this reason, we opted to investigate the involvement of C3.

CD47, an integrin-associated protein, has been described as a “don’t eat me” signal, having a protective role of VGluT2^+^ synapses—but no effect on VGluT1^+^ type–in the visual system (Lehrman et al., 2018). However, another study revealed that CD47 affected VGluT1 synapses in the hippocampus (Ding et al., 2021), rendering the function of CD47 less defined. Furthermore, under pathological conditions, tumor cells overexpress CD47 to avoid engulfment by macrophages (Willingham et al., 2012). In contrast, studies in cortical and hippocampal regions have reported that elimination of SIRPα, the CD47 receptor, contributes to synapse elimination at late but not early stages of development (Ding et al., 2021). Thus, the function of CD47 in synapse elimination is currently unclear.

To elucidate the molecular mechanisms of synaptic elimination and their potential role in the refinement of sensory-motor circuits during early development, we investigated whether activation of the classical complement cascade is causally involved. Specifically, we investigated the involvement of classical complement proteins C1q and C3 in spinal sensory-motor circuits during postnatal mouse development. We also studied whether CD47 acts as a protecting agent for synapses. To address these questions, we used the well-established proprioceptive-motor neuron circuit responsible for the stretch reflex in the hindlimb of neonatal mice. Using virally mediated neuronal map strategies, physiology, behavioral assays, and mouse genetics to eliminate ubiquitously C1q, C3, and CD47, either alone or in combination, we systematically investigated the cascade of events involved in the formation and function of sensory-motor spinal circuits.

In both C3 and C1q knockout mice, we observed a robust increase of proprioceptive synapses within the motor neuron pools during early postnatal ages. Remarkably, we also observed a similar result in CD47 knockout mice, in contrast to its neuroprotective function in the brain. We determined that this aberrant increase (∼30%) of proprioceptive synapses in mutant mice resulted in inappropriate synaptic contacts between flexor proprioceptors and antagonistic (extensor) motor neurons, and vice versa. These inappropriate synapses were functional and responsible for altered functional characteristics in motor neurons, leading to an abnormal behavioral phenotype. Taken together, our results identify two distinct molecular mechanisms —C3 through classical complement activation and CD47-SIRPα interactions—that mediate engulfment and removal of inappropriate synapses by microglia within spinal sensory-motor circuits. Importantly, we identified a previously unknown role for CD47 being involved in synaptic elimination in the spinal cord.

## Results

### Genetic deletion of C3 or CD47 results in supernumerary proprioceptive synapses

To investigate whether the classical complement pathway is involved in the elimination of supernumerary synapses in the developing spinal cord, we examined the role of C3 protein, a key regulator protein, downstream of C1q, in the activation of the classical complement pathway (Presumey et al. 2017; Schafer et al., 2012). At the same time, we investigated the potential protective role of CD47, since it has been reported to play a "don’t eat me” role for synapses in the retinogeniculate system (Lehrman et al., 2018).

**Figure 1.**
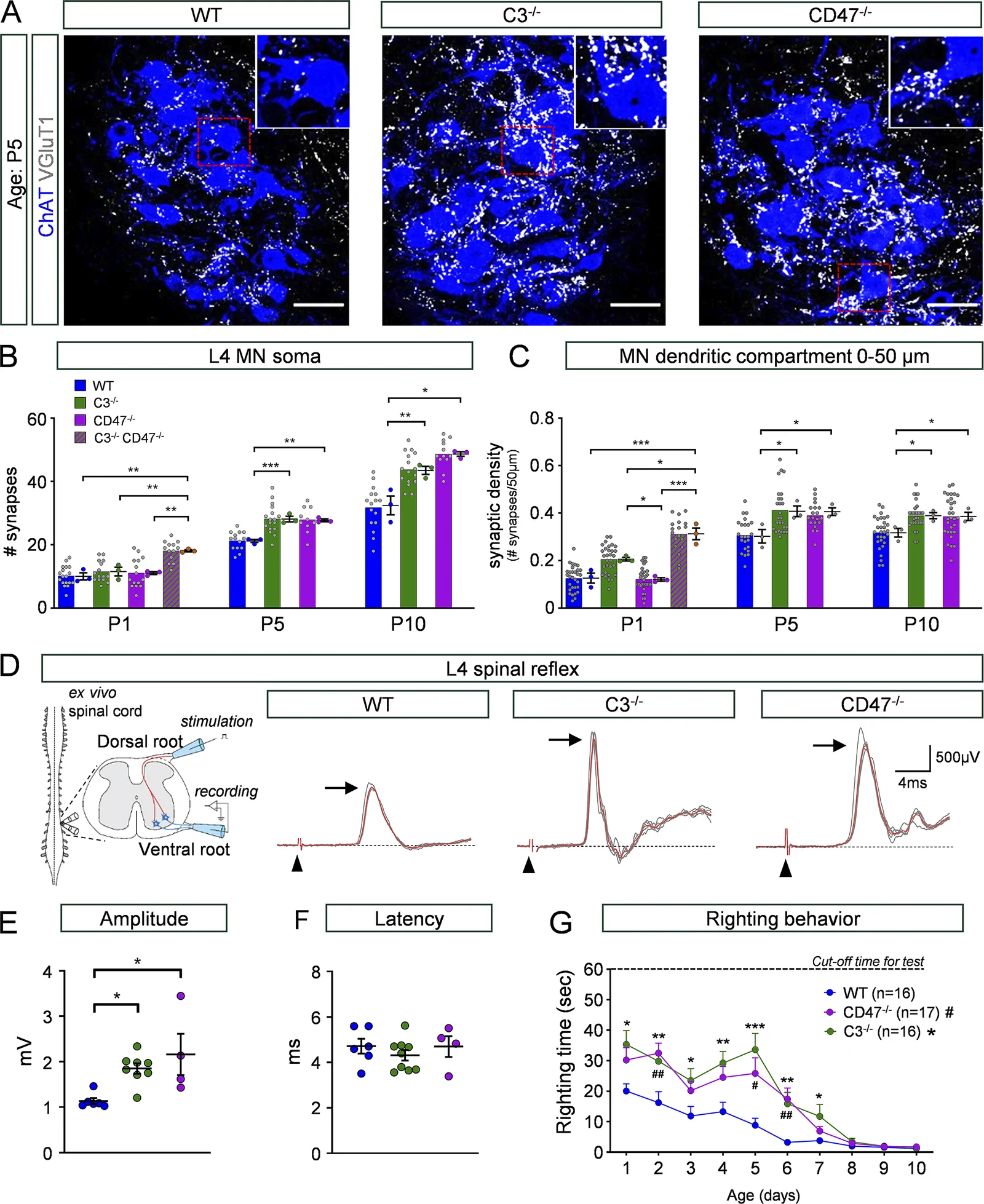
**Genetic deletion of C3 or CD47 results in a higher incidence of proprioceptive (VGlut1^+^) synapses on spinal motor neurons. (A)** Confocal images of L4 motor neurons (ChAT, blue) and VGluT1 synapses (VGluT1, white) in WT, C3^−/−^, and CD47^−/−^ mice at P5. Insets show higher magnification images of a single motor neuron. Scale bar: 50 μm. Images are Z-stack projection 3.5 μm in total distance (at 0.35 μm intervals). **(B)** Number of VGluT1 synapses per motor neuron soma at P1, P5, and P10 (*N* = 3 mice/group; colored data points). Number of MN somata analyzed: (WT: P1, *n* = 18; P5, *n* = 14; P10, *n* = 16) (C3^−/−^: P1, *n* = 18; P5, *n* = 17; P10 *n* = 16) (CD47^−/−^: P1, *n* = 15; P5, *n* = 10; P10, *n* = 12) are shown in grey data points and bar graphs from all mice per group. **(C)** Synaptic density of proximal dendrites (0–50 μm from the soma, *N* = 3 mice/group). Number of MN dendrites analyzed: (WT: P1, *n* = 30; P5, *n* = 24; P10, *n* = 30) (C3^−/−^: P1, *n* = 30; P5, *n* = 30; P10, *n* = 30) (CD47^−/−^: P1, *n* = 30; P5, *n* = 20; P10, *n* = 30). Significance: *P < 0.05, **P < 0.001, ***P < 0.001; one-way ANOVA with multiple comparisons using Bonferroni’s test. Statistical comparison was performed for average values in mice (colored data points). **(D)** Simplified schematic of the ex vivo spinal cord preparation to assess the dorsal-ventral reflex at P5. Representative recordings of L4 ventral root responses following L4 dorsal root stimulation in WT, C3^−/−^, and CD47^−/−^ mice. The red trace is the average of the first five trials, which are shown in grey. Arrowheads indicate stimulus artifacts. Black arrows indicate peak amplitude in the averaged response. **(E)** Maximum amplitude of spinal reflexes at P5 (WT: *N* = 6 mice; C3^−/−^: *N* = 8; CD47^−/−^: *N* = 4). Significance: *P < 0.05, one-way ANOVA with multiple comparisons using Bonferroni’s test. **(F)** Latency of responses for the same group as in E. **(G)** Righting times across the first 10 postnatal days for WT, C3^−/−^, and CD47^−/−^ mice. Significance: (*) (#) P < 0.05, (**) (##) P < 0.01, and (***) P < 0.001 (compared with WT: CD47 significance #; C3 significance *), one-way ANOVA with multiple comparisons using Bonferroni’s test. MN, motor neuron.

**Figure S1.**
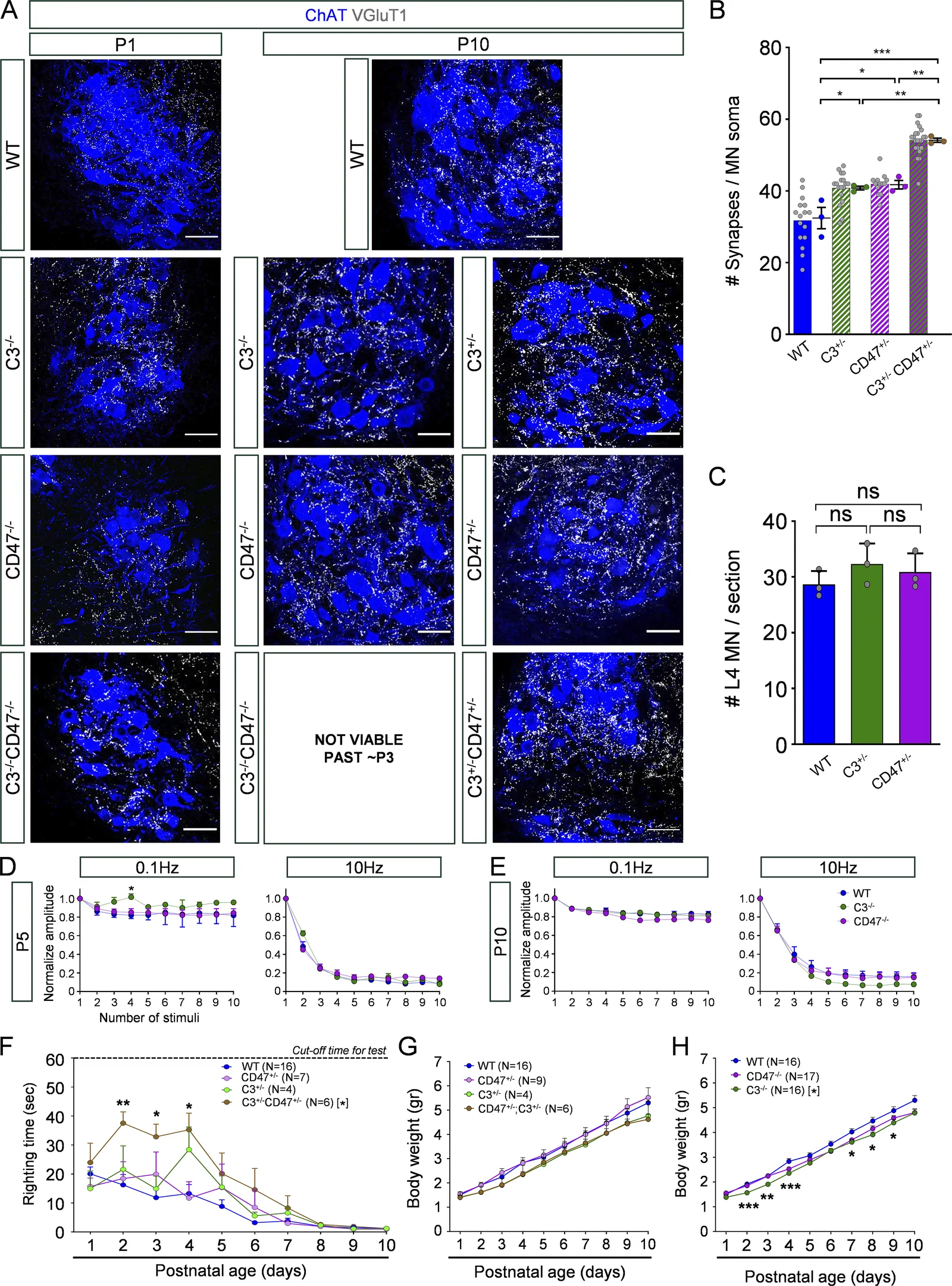
**Effects of genetic deletion of C3 alone, CD47 alone, or combined in the sensory-motor circuit. (A)** Single optical plane confocal images of L4/5 spinal cord ventral horns from WT, C3^−/−^, CD47^−/−^, and C3^−/−^CD47^−/−^ mice at P1 (left column) and WT, C3^−/−^, C3^+/−^, CD47^+/−^, CD47^−/−^, and C3^+/−^CD47^+/−^ at P10 (middle and right columns), with antibodies against ChAT (blue) and VGlut1 (white). Double homozygous knockout mice for C3 and CD47 (C3^−/−^CD47^−/−^) were not viable after ∼P3. Scale bar: 50 μm. **(B)** Number of VGluT1 synapses per motor neuron soma in WT, C3^+/−^, CD47^+/−^ (hets) and C3^+/−^CD47^+/−^ (double het) mice at P10 (*N* = 3 mice/group). Number of motor neuron somata analyzed: WT: *n* = 17 MNs; C3^+/−^: *n* = 16; CD47^+/−^: *n* = 13; C3^+/−^CD47^+/−^: *n* = 22 (grey data points), from *N* = 3 mice/group (colored data points). Significance: *P < 0.05, **P < 0.001, and ***P < 0.001; one-way ANOVA, Tukey’s post hoc test. **(C)** Number of L4 motor neurons per 75-μm-thick section for three spinal cord sections per animal from WT (*N* = 3), C3^−/−^ (*N* = 3), and CD47^−/−^ (*N* = 3) mice at P5. Quantification was performed from a single side of the spinal cord. **(D)** Normalized (with respect to first response) amplitude change of spinal reflex challenged at 0.1 and 10 Hz in WT (blue: 0.1 Hz, *N* = 4 mice; 10 Hz, *N* = 3), C3^−/−^ (green, 0.1 Hz, *N* = 4; 10 Hz, *N* = 4), and CD47^−/−^ mice (purple: 0.1 Hz, *N* = 4; 10 Hz, *N* = 4) at P5. Significance: C3^−/−^ versus WT: *P < 0.05; two-way ANOVA multiple comparisons with Bonferroni’s test. **(E)** Same analysis as in E but at P10. WT (blue, 0.1 Hz, *N* = 8; 10 Hz, *N* = 8), C3^−/−^ (green: 0.1 Hz, *N* = 4; 10 Hz, *N* = 4), and CD47^−/−^ (purple, 0.1 Hz, *N* = 3; 10 Hz, *N* = 3). No significant difference was observed; two-way ANOVA with multiple comparisons using Bonferroni’s test. **(F)** Righting time for WT (*N* = 16 mice), C3^+/−^ (*N* = 4), CD47^+/−^ (*N* = 7), and C3^+/−^CD47^+/−^ (*N* = 6) animals from P1 until P10. **(G)** Body weight gain in WT (*N* = 16), CD47^+/−^ (*N* = 9), C3^+/−^ (*N* = 4), and C3^+/−^CD47^+/−^ (*N* = 6) mice. No significance was observed; one-way ANOVA with multiple comparisons using Bonferroni’s test. **(H)** Body weight gain in WT (*N* = 16), CD47^−/−^ (*N* = 17), C3^−/−^ (*N* = 16), and C3^+/−^CD47^+/−^ (*N* = 6) mice. Significance: C3^−/−^ versus WT: *P < 0.05, **P < 0.01, ***P < 0.001; one-way ANOVA with multiple comparisons using Bonferroni’s test. MN, motor neuron.

To address the involvement of C3 and CD47 in synaptic regulation in the developing spinal cord, we assessed proprioceptive synapses (marked by VGluT1 and opposed on motor neurons) in the fourth or fifth lumbar (L4 or L5) spinal segments during early postnatal development in WT, C3 knockout (C3^−/−^, homozygous knockout), CD47 knockout (CD47^−/−^, homozygous knockout), and C3+CD47 double knockout (C3^−/−^CD47^−/−^, homozygous knockout) mice. The somatic and dendritic (0–50 μm distance from the soma) coverage of motor neurons (ChAT^+^ located in the ventral horn) by proprioceptive synapses was analyzed by immunohistochemistry and quantified using confocal microscopy at the postnatal ages, P1, P5, and P10 (Fig. 1 A and Fig. S1, A and B). The double homozygous knockout mice C3^−/−^CD47^−/−^ were viable only for a few days after birth (survived until ∼P2/3). We found that the proprioceptive synaptic coverage increased with age in WT mice (blue bars in Fig. 1, B and C). At P1, there was no significant difference between WT, C3^−/−^, and CD47^−/−^ mice. In contrast, C3^−/−^CD47^−/−^ mice revealed a significantly higher incidence of synapses on both soma and dendrites (Fig. 1, B and C; and Fig. S1 A) of motor neurons compared with WT mice. Importantly, we observed a significantly higher incidence of proprioceptive synapses in C3^−/−^ mice at P5 and P10 compared with their age-matched counterparts (Fig. 1, B and C). Remarkably, CD47^−/−^ mice also revealed a higher incidence of proprioceptive synapses, similar to that observed in C3^−/−^ mice (Fig. 1, A–C).

To investigate whether combined knock out of C3 and CD47 would result in a greater increase of synapses, we compared single and double heterozygous mice with their WT counterparts at P10, since double homozygous mice do not survive past P2/3. We found that heterozygous C3^+/−^ (Fig. S1, A and B) or heterozygous CD47^+/−^ (Fig. S1, A and B) mice exhibit a significantly higher number of synapses on motor neuron somata at P10 compared with WT, similar to the homozygous knock out. This finding indicates that homozygosity of either C3 or CD47 knockout does not result in a higher incidence of synapses. However, combined heterozygous mice (C3^+/−^CD47^+/−^) revealed a significantly higher incidence of synapses compared with individual heterozygous mice (Fig. S1, A and B).

We next sought to investigate whether the supernumerary proprioceptive synapses are functional in both C3^−/−^ and CD47^−/−^ mice. To address this, we performed physiological experiments in which proprioceptive synapses were tested using the ex vivo spinal cord. We quantified the amplitude of the L4 dorsal-to-ventral root spinal reflex using the ex vivo spinal cord preparation (Fig. 1 D), as we previously described (Mentis et al., 2011; Vukojicic et al., 2019). We found that the L4 reflex amplitude was significantly increased in C3^−/−^ and CD47^−/−^ mice compared with their WT counterparts at P5 (Fig. 1, D and E). The latency of the monosynaptic responses, however, did not show any significant difference among the three experimental groups (Fig. 1 F). These results demonstrate that the supernumerary synapses are functional in both C3^−/−^ and CD47^−/−^ mice. We also investigated whether genetic deletion of C3 or CD47 would impact the number of spinal motor neurons but found no significant difference in the average number of L4 motor neurons (Fig. S1 C). Next, to test whether excessive proprioceptive synapses are susceptible to synaptic depression, we challenged these synapses at two different frequencies (0.1 and 10 Hz). We found no differences in synaptic depression among the three groups, at both P5 and P10 (Fig. S1, D and E). This result indicates that supernumerary synapses do not differ in neurotransmission reliability compared with WT counterparts.

To investigate the impact of an excessive number of synapses on mouse behavior, we quantified the righting time in mice and found significantly longer response times in both C3^−/−^ and CD47^−/−^ mice compared with WT controls in the first postnatal week (Fig. 1 G and Video 1). To this end, double heterozygous mice for C3 and CD47 (C3^+/−^CD47^+/−^) also exhibited longer latencies to right (Fig. S1 F, Video 2), especially during the first 5 days after birth, when proprioception plays a major role in this behavior (Fletcher et al., 2017). While heterozygous mice did not exhibit any significant body weight gain (Fig. S1 G), homozygous mice for C3 (C3^−/−^) or CD47 (CD47^−/−^) revealed some mild differences in body weight gain (Fig. S1 H).

**Video 1.** Righting time for a WT (as control) mouse, a C3 knockout, and a CD47 knockout mouse (age of all mice: P5).

**Video 2.** Righting time for a WT mouse (as control), a C3/CD47 double heterozygous mouse, as well as a C3 heterozygous and a CD47 heterozygous mouse (age of all mice: P5).

**Figure 2.**
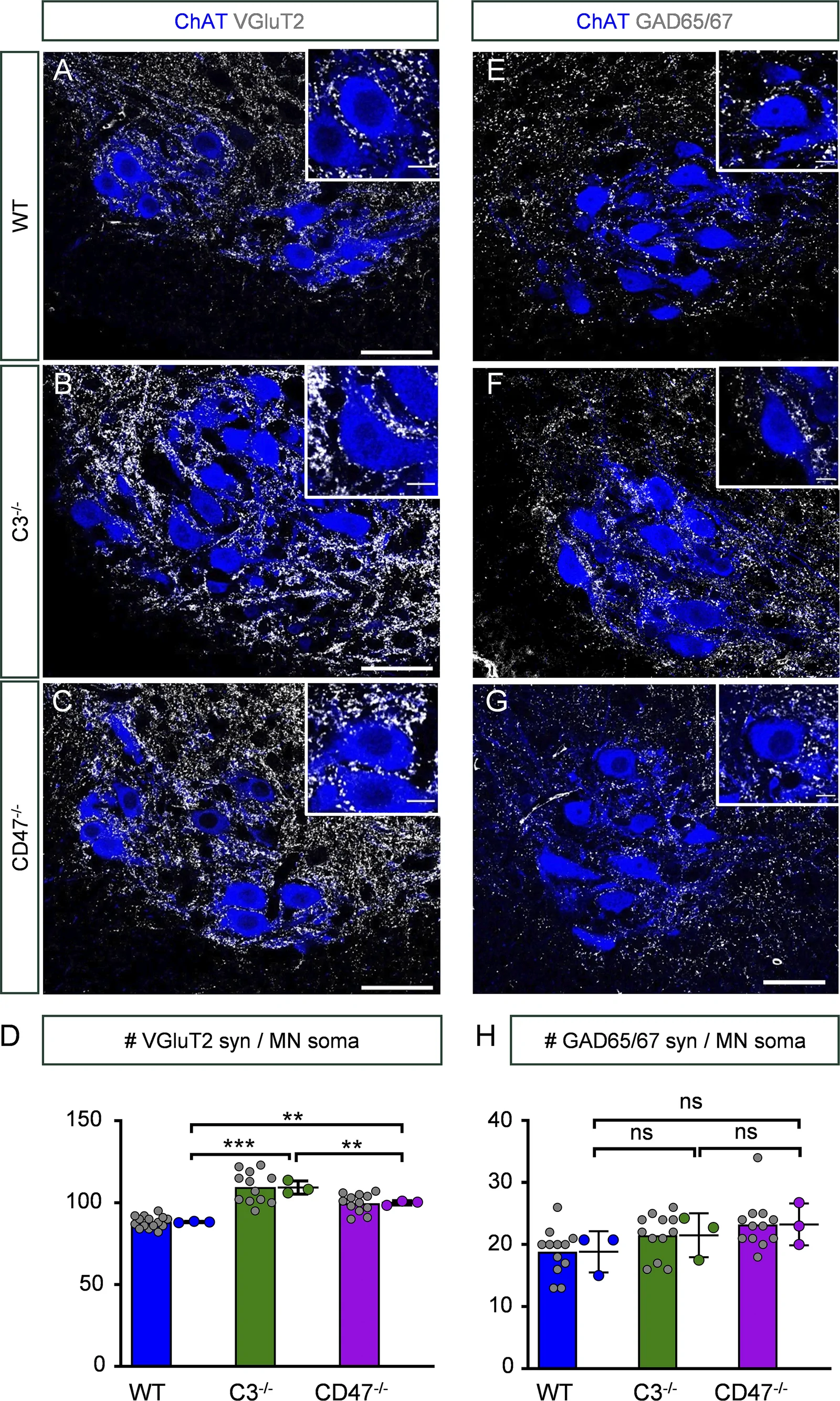
**Effects of genetic deletion of C3 and CD47 on excitatory VGluT2 and inhibitory GAD65/67 synaptic refinement. (A–C)** Single optical plane confocal images from L4 motor neurons at P5. Immunoreactivity for ChAT (blue) and VGluT2^+^ (white) synapses in WT, C3^−/−^ (B), and CD47^−/−^ (C) mice (scale bars in A, B, and C are all 50 μm). Insets show higher magnification of a single motor neuron with VGluT2^+^ synapses (scale bar: 10 μm). **(D)** Quantification of VGluT2^+^ synapses on the entire motor neuron soma for each genotype. Data points (in grey) on graphs show individual motor neurons; colored data points represent the average value from each mouse. **(E–G)** Single optical plane confocal images from L4 motor neurons at P5. Immunoreactivity for ChAT (blue) and GAD65/67^+^ (white) synapses in WT, C3^−/− ^(F), and CD47^−/−^ (G) mice (scale bars in E, F, and G are 50 μm). Insets show higher magnification of a single motor neuron with GAD65/67^+^ synapses (scale bar: 10 μm). **(H)** Quantification of GAD65/67^+^ synapses on the entire motor neuron soma for each genotype. Data points (in grey) on graphs show individual motor neurons; colored data points represent the average of four to five motor neurons from each mouse. Statistical comparison was performed across mouse averages. WT, *N* = 3 mice; C3^−/−^, *N* = 3; CD47^−/−^, *N* = 3. **P < 0.01, ***P < 0.001; ns: no significance; one-way ANOVA, post hoc Bonferroni’s test.

We next investigated whether the effect of C3 and CD47 deletion was restricted to proprioceptive synapses only or would impact other synapses in the spinal cord. We performed experiments in both C3^−/−^ as well as CD47^−/−^ and compared the results with those obtained from WT mice. We quantified the number of VGluT2^+^ (excitatory; Fig. 2, A–D) or GAD65/67^+^ (inhibitory; Fig. 2, E–H) synapses on the entire motor neuron soma for four to five motor neurons per mouse, identical to the VGluT1 assessment. We found that both C3 and CD47 mediate the reduction of VGluT2^+^ (Fig. 2 D), but not GAD65/67^+^ synapses (Fig. 2 H).

Taken together, these results demonstrate that supernumerary excitatory synapses form in the spinal cord during early development and suggest that both classical complement C3-dependent and C3-independent mechanisms are responsible for the elimination of proprioceptive as well as other excitatory synapses, but not inhibitory synapses. Furthermore, it indicates that CD47 might participate in synaptic removal, playing a surprisingly different and opposing role in the spinal cord compared with the brain.

### Supernumerary synapses are largely inappropriate and are eliminated via C3 or CD47 activity

Deletion of C3 or CD47, resulting in a higher incidence of proprioceptive synapses, raised the possibility that these synapses may be inappropriately formed. Inappropriate synapses are considered those between sensory fibers originating from a flexor muscle and contacting an antagonistic motor neuron innervating an extensor muscle, or vice versa. If this hypothesis is correct, inappropriate synapses are destined to be eliminated during early development as part of a refinement process and the emergence of mature sensory-motor circuits. To establish whether deletion of C3 or CD47 induces formation of inappropriate synapses, we investigated the possibility that proprioceptive fibers that innervate the dorsiflexor muscle tibialis anterior (TA) make inappropriate contacts with motor neurons that innervate the biomechanically antagonistic muscle gastrocnemius (Gs; plantar extensor muscle) or vice versa. To determine this, we utilized a virally-mediated mapping strategy combined with genetically modified mice, similar to that described previously (Balaskas et al., 2019). Specifically, we used a PV:FlpO allele (targeting proprioceptors) (Celio 1990; de Nooij et al. 2013; Ichikawa et al., 1994; Oliver et al., 2021) and Cre and FlpO-dependent TdTomato reporter mice (Ai65:tdT) to label select proprioceptive fibers and their synapses following muscle injections of a virally Cre-expressed driver. To this end, PV::FlpO were crossed with the Ai65 Cre/FlpO-dependent TdTomato reporter, and the resulting animals (Pv::FlpO^+/+^; Ai65^+/+^) were crossed with C3^−/−^, C1q^−/−^ and C47^−/−^ mouse lines to obtain *Pv::FlpO*^*+/+*^*/Ai65*^*+/+*^/*C3*^−/−^; *Pv::FlpO*^*+/+*^/*Ai65*^*+/+*^*/C1q*^−/−^; *Pv::FlpO*^*+/+*^*/Ai65*^*+/+*^*/CD47*^*−/−*^ strains, respectively.

**Figure 3.**
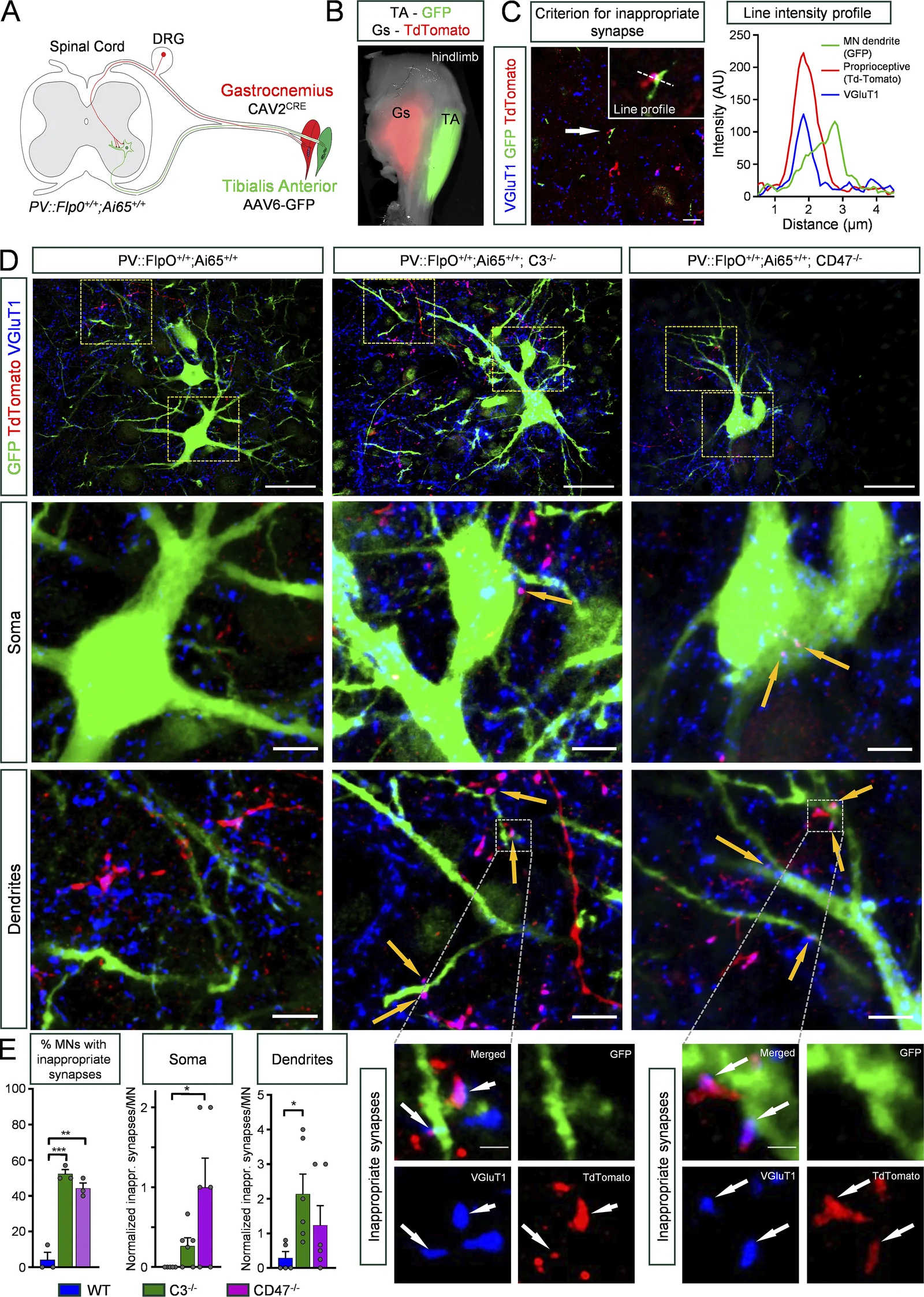
**Inappropriate synapses on motor neurons formed by proprioceptive fibers originate in antagonistic muscles in C3** ^**−/−**^ **and CD47** ^**−/−**^ **mice. (A)** Schematic revealing the strategy for labelling proprioceptive fibers originating in Gs muscle and labelling of motor neurons innervating the antagonistic TA muscle. TA motor neurons were labelled by injection of AAV6-GFP in TA muscle, and Gs proprioceptors were labelled by injection of CAV2^CRE^ virus in the Gs muscle in PV::FlpO;Ai65 mice. Injections were performed at P0/1, and tissue was harvested at P6/7. **(B)** Superimposed fluorescent and transmitted-light photograph of the hindlimb injected at P5, showing the Gs muscle in red (TdTomato) and the TA muscle in green (GFP). **(C)** Confocal image of an inappropriate synapse. Inset shows the line profile across the synapse (white arrow in low-magnification image), which was used as our criterion for synaptic contact. Graph shows the signal intensity profile for each of the three immunoreactive signals. Gs proprioceptive fibers labelled with DsRed (red), TA MN dendrites (green), and VGlut1 synapses (blue). **(D)** Z-stack projection confocal images of L4 TA motor neurons from PV::FlpO;Ai65 (left), PV::FlpO;Ai65;C3^−/−^ (middle), and PV::FlpO;Ai65;CD47^−/−^ (right) mice. Z-stack distance: 3.5 μm. Top row, scale bar: 100 μm. Magnified image from the yellow dotted box in the soma (center row) and dendrite (bottom row). Yellow arrows point to inappropriate synapses. Scale bar: 20 μm. Higher magnification insets from white dotted boxes showing inappropriate synapses on dendrites (bottom row) in PV::FlpO;Ai65;C3^−/−^ and PV::FlpO;Ai65;CD47^−/−^ mice only. White arrows highlight inappropriate synapses. Scale bar: 2 μm. **(E)** Percentage of motor neurons with inappropriate synapses (on soma and dendrites). Left graph, WT: *N* = 3 mice; C3^−/−^: *N* = 3; CD47^−/−^: *N* = 3 (age: P5–P7). Middle and right graphs: normalized number of inappropriate somatic (center) and dendritic (right) synapses per motor neuron (WT: *N* = 5; C3^−/−^: *N* = 6; CD47^−/−^: *N* = 6 sections from three mice per group). Significance: *P < 0.05, **P < 0.001, and ***P < 0.001; one-way ANOVA with multiple comparisons using Bonferroni’s test. MN, motor neuron.

**Figure S2.**
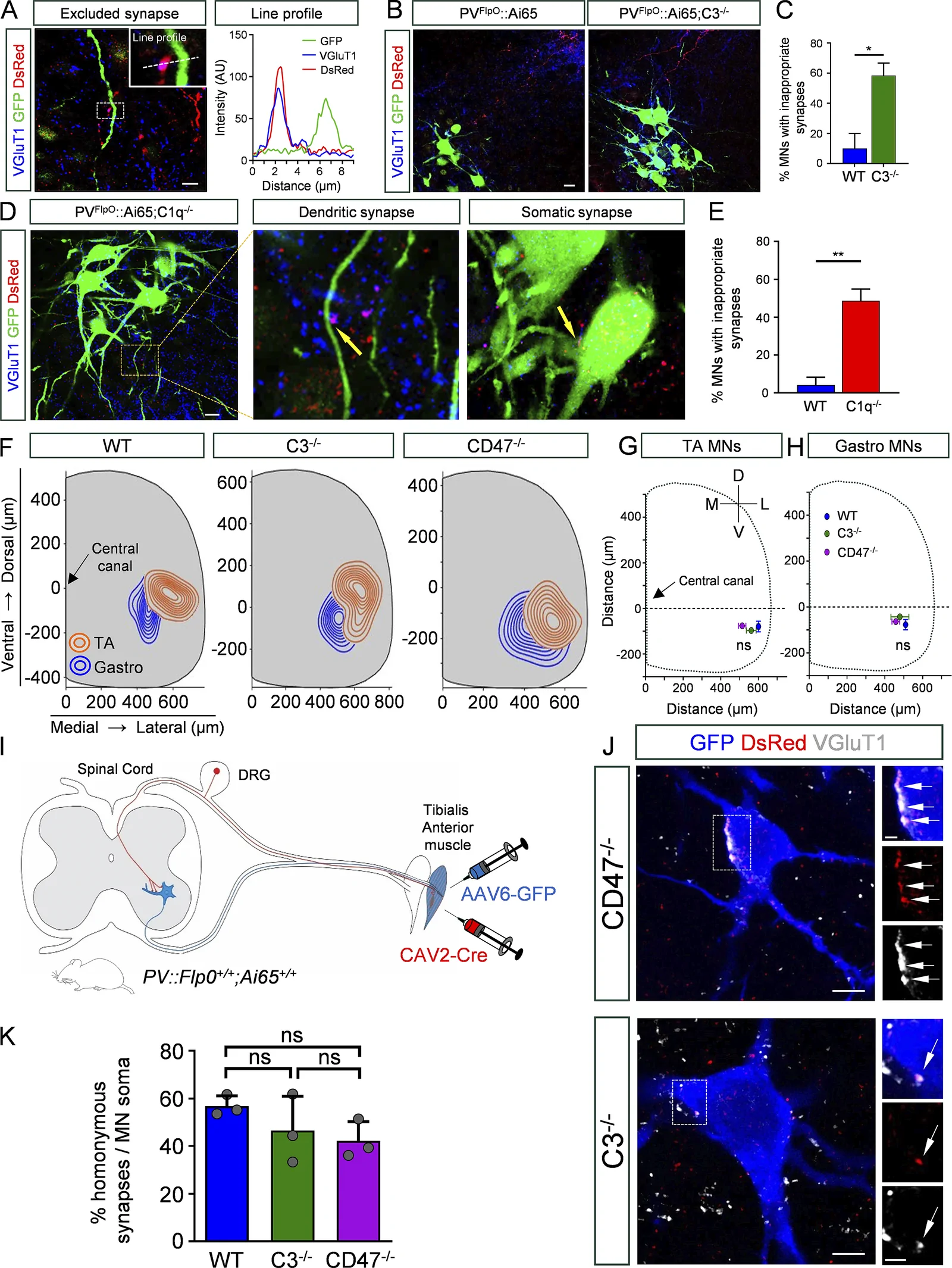
**Exclusion criterion for inappropriate synapses that are formed between TA proprioceptors and Gs motor neurons, density maps for TA and Gs motor neuron pools, and no change in appropriate synapses in C3** ^**−/−**^ **and CD47** ^**−/−**^ **mice. (A)** Criterion for exclusion of synapses from our analysis. Image depicting an excluded synapse labelled in DsRed (red) and colocalized with VGluT1 (green) because it was not opposed to a dendrite of a TA motor neuron (shown in green; see graph where there is no overlap between green and those in red and blue) in a P5 PV::FlpO;Ai65 mouse at P5. Scale bar: 10 μm. **(B)** Z-stack confocal images (total: 3.5 μm) from a Gs motor neuron pool in PV::FlpO;Ai65 and PV::FlpO;Ai65;C3^−/−^ mouse injected with rAAV6-GFP virus in Gs muscle and CAV2^CRE^ in the TA muscle. Scale bar: 20 μm **(C)** Percentage of motor neurons (MNs) with inappropriate synapses from TA proprioceptors to Gs motor neurons. Significance: *P < 0.05; unpaired *t* test. **(D)** Identification of inappropriate synapses in PV::FlpO;Ai65;C1q^−/−^ mice, labelled by DsRed (red) and VGluT1 (blue) on TA motor neurons (GFP; green). Yellow arrows show dendritic and somatic inappropriate synapses in higher magnification images. Scale bar: 20 μm. **(E)** Percentage of motor neurons with inappropriate synapses in WT and C1q^−/−^ mice (WT, *N* = 3 mice; C1q^−/−^, *N* = 4). **P < 0.001; unpaired *t* test. **(F)** Density maps for TA (red) and Gs (blue) motor neuron pools in WT, C3^−/−^, and CD47^−/−^ mice. Maps represent the ventro-dorsal and mediolatearal distribution of MNs between L4 and L5 (450 μm total). The value 0 in y-axis represents the central canal. **(G and H)** Quantification of “hot area” distribution of each type of motor neuron, TA (G) and Gs (H). ns: no significance, one-way ANOVA with multiple comparisons using Bonferroni’s test. D, dorsal; V, ventral; M, medial; L, lateral. **(I)** Schematic illustration of experimental design. The TA muscle was co-injected with AAV6-GFP (blue) and CAV2-Cre (red) viruses using PV::FlpO^+/+^;Ai65^+/+^ mice at birth (P0) and the spinal cord was examined at P5. **(J)** Single optical plane confocal images from L4 motor neurons at P5 in CD47^−/−^ (top) and C3^−/−^ (bottom) mice. Immunostaining against GFP (blue), homonymous proprioceptive synapses (amplified by an antibody against DsRed; immunoreactive signal is shown in red) and VGluT1^+^ synapses (white). GFP labels TA motor neurons only. DsRed labels the fibers and synapses of proprioceptive neurons originating in the TA muscle. Fluorochrome-separated images are shown for clarity for DsRed only and VGluT1^+^ only synapses, as well as merged images (motor neurons are GFP^+^, proprioceptive fibers/synapses are DsRed^+^, VGluT1 for synapses only) (scale bar: 10 μm). Insets represent higher magnification images of individual synapses; white arrows indicate colocalization between DsRed and VgluT1^+^ signals (inset scale bars: 4 μm). **(K)** Percentage of homonymous proprioceptive synapses (VGluT1^+^ and DsRed^+^) per TA motor neuron soma, relative to the total number of VGluT1^+^ synapses per motor neuron soma. At least five TA motor neurons were examined per mouse. WT, *N* = 3 mice; C3^−/−^, *N* = 3; CD47^−/−^, *N* = 3. ns: no significance; one-way ANOVA, post hoc Bonferroni’s test.

To activate expression of the fluorescent tdTomato reporter in Gs proprioceptors, we injected a canine adeno virus 2 expressing Cre (CAV2^CRE^) (Chalif et al., 2022) into the Gs muscle at P0 (Fig. 3 A). In addition, we simultaneously injected an rAAV6/ds-CBH-GFP (AAV6-GFP) virus in the TA muscle to drive expression of GFP and label the soma and dendrites of TA motor neurons (Fig. 3, A and B). Next, we investigated putative inappropriate synapses between Gs-originated proprioceptors and TA motor neurons at P6/7. Our criterion of an inappropriate synapse required colocalization of TdTomato in a Gs-proprioceptor fiber with VGluT1 immunoreactivity, which, in turn, was opposed to a soma or dendrite of a TA motor neuron (labelled with GFP). This criterion was met through line intensity profiling (Fig. 3 C) from single optical plane confocal images (see Materials and methods for more details). For quantification purposes, only VGluT1 (proprioceptive synaptic marker) and TdTomato (proprioceptive fiber/synapse originating from an identified muscle) signals partially overlapping with the GFP (motor neuron) signal were considered inappropriate synapses (Fig. 3 C). If the colocalized TdTomato and VGluT1 signals were not opposed to GFP (Fig. S2 A), these synapses were excluded from analysis. Under such stringent criteria, we found a nearly complete absence of inappropriate synapses on either the soma or proximal dendrites of TA motor neurons in WT mice (Fig. 3, D and E). In striking contrast, ∼55% of TA motor neurons in C3^−/−^ and ∼45% of TA motor neurons in CD47^−/−^ mice exhibited a significantly higher number of inappropriate synapses (Fig. 3, D and E). Additionally, we observed that the location of inappropriate synapses was different in each of the two mutant mouse lines. CD47^−/−^ mice revealed a significant increase in inappropriate synapses on the soma of TA motor neurons, while C3^−/−^ animals exhibited more inappropriate synapses on proximal dendrites (Fig. 3, D and E).

We then investigated whether there is a preferential direction for inappropriate proprioceptive synapses originating from dorsiflexor muscles (i.e., TA muscle) to plantar extensor (i.e., Gs) motor neurons. To address this possibility, we reversed the injections of the two viruses injected (CAV2^CRE^ in TA and AAV6-GFP in Gs). Our analysis revealed that inappropriate synapses were also formed between TA proprioceptors and Gs motor neurons in C3^−/−^ mice to a similar extent (Fig. S2, B and C). To provide further support that the classical complement cascade is involved in synaptic refinement, we examined whether spinal motor neurons receive inappropriate synapses in C1q knockout mice. C1q is the initiating protein in the classical cascade pathway. As expected, C1q knockout mice revealed ∼50% of motor neurons with inappropriate synapses (Fig. S2, D and E).

We also investigated whether the inappropriate synapses in C3^−/−^ and CD47^−/−^ mice might be the result of scrambled motor neuron position within the ventral horn, since previous reports demonstrated that when motor neuron position is altered, the proper organization of sensory-motor circuits is lost (Sürmeli et al., 2011; Balaskas et al., 2019). To address this possibility, TA and Gs motor neurons were selectively labelled with two different fluorescent tracers injected from their corresponding muscle (CTb488 in TA and CTb555 in Gs) at P0. At P5, spinal cord sections containing L4/L5 lumbar segments were scanned and mapped with Neurolucida (see Materials and methods), and density maps were constructed indicating the dorsoventral and mediolateral extent of both TA and Gs motor neuron pools in WT, C3^−/−^, and CD47^−/−^ mice (Fig. S2 F). Using the central canal as a reference point, the contour of the transverse area in which TA and Gs motor neurons were positioned within the ventral horn according to the coordinates was identified, mapped, and statistically compared. We found no significant differences in the density map of the location for either TA motor neurons (Fig. S2 G) or Gs motor neurons (Fig. S2 H and Video 3) in all three experimental groups.

**Video 3.** The location of TA and Gs motor neurons in 3D space (rendered from individual single optical planes from confocal images) from the L4/L5 spinal segments of a C3 knockout mouse (D, dorsal; V, ventral; A, anterior; P, posterior; L, lateral; CC, central canal).

**Figure 4.**
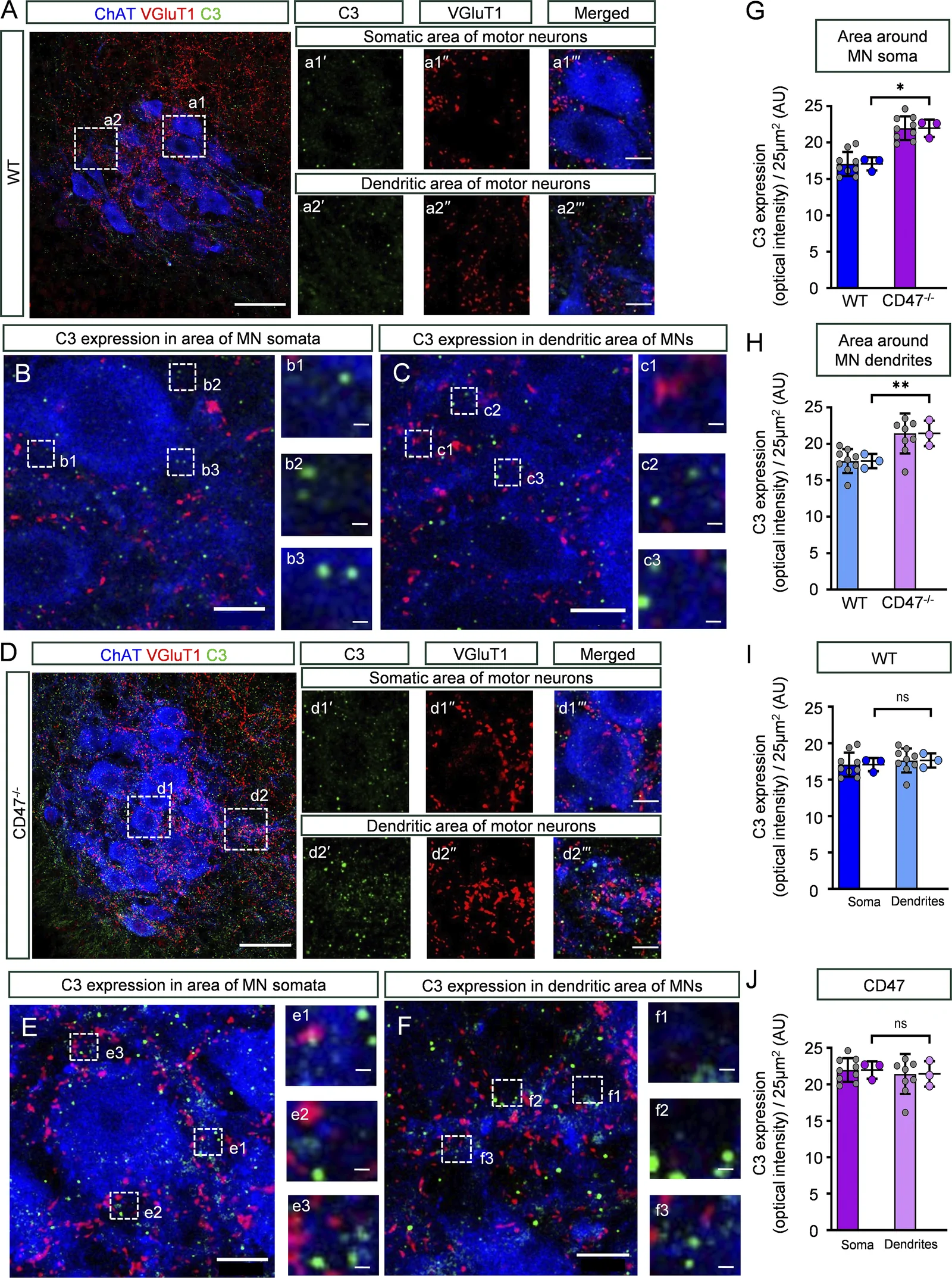
**C3 expression in WT and CD47** ^**−/−**^ **spinal cords. (A)** Single optical plane confocal images from L4 motor neurons at P5 in WT mice. Immunoreactivity for ChAT (blue), VGluT1 (red), and C3 (green) (scale bar: 50 μm). Insets (a1 and a2) depict examples from somatic and dendritic areas, respectively. a1′ and a2′ = C3 (green), a1″ and a2″ = VGluT1 (red), and a1‴ and a3‴ = merged with ChAT (scale bar: 10 μm). **(B and C)** Higher magnification of C3 protein expression around motor neuron somata and (C) around motor neuron dendrites (scale bar: 10 μm). Insets b1–b3 depict C3 expression surrounding motor neuron soma, while c1–c3 correspond to dendritic areas (scale bar: 1 μm). **(D)** Single optical plane confocal images from L4 motor neurons at P5 in CD47^−/−^ mice. Immunoreactivity for ChAT (blue), VGluT1^+^ synapses (red), and C3 (green) (scale bar: 50 μm). Insets (d1 and d2) depict examples from somatic and dendritic areas, respectively. d1′ and d2′ = C3 (green), d1″ and d2″ = VGluT1 (red), and d1‴ and d2‴ = merged with ChAT (scale bar: 10 μm). **(E and F)** (E) Higher magnification of C3 protein expression around motor neuron somata and (F) around motor neuron dendrites (scale bar: 10 μm). Insets e1–e3 depict C3 expression surrounding motor neuron soma, while f1–f3 correspond to dendritic areas (scale bar: 1 μm). All images (A–F) were acquired under identical scanning conditions. **(G–J)** Quantification of optical intensity of C3 expression from areas of motor neuron somata, and dendrites in WT and CD47^−/−^ mice. Data points in bar charts were from three images (area: 25 μm^2^) per animal (shown in B and E images for somata and C and F images for dendrites). Colored data points are averages from individual mice. **(G–J)** (G) Somata: WT versus CD47^−/−^; (H) dendrites: WT versus CD47^−/−^; (I) WT: somata versus dendrites; (J) CD47^−/−^: somata versus dendrite. Three images were analyzed per animal. WT, *N* = 3 mice; CD47^−/−^, *N* = 3 mice. Statistical comparison was performed between averages from individual mice. ns: no significance; *P < 0.05, **P < 0.01. Unpaired *t* test.

We next wanted to address whether the localization of inappropriate synapses on the somata versus the dendrites of motor neurons (as shown in Fig. 3 E) correlate with the presence/absence of C3 or CD47 in those areas. To address this, we performed an optical intensity quantification of C3 expression following immunoreactivity experiments for ChAT (to label motor neurons), VGluT1 (to label synapses), and C3, around motor neuron somata and compared C3 intensity with that obtained around dendrites in WT (Fig. 4, A–C) and CD47^−/−^ (Fig. 4, D–F) mice. We found a significant increase of C3 signal in CD47^−/−^ mice compared with WT controls in both somatic and dendritic areas of motor neurons (Fig. 4, G and H). However, there was no significant difference in C3 expression around somata compared with dendrites in WT (Fig. 4 I) and CD47^−/−^ mice (Fig. 4 J). This result indicates that the expression of C3 does not differ along the motor neuron area, even though the presence of inappropriate synapses in C3^−/−^ mice is more evident in dendrites. Taken together, these results show that there is no correlation of C3 expression with the increased elimination of inappropriate synapse.

Lastly, to test whether C3 and CD47 impact inappropriate synapses only, we quantified the appropriate proprioceptive synapses, which originate from a single muscle and synapse on their homonymous motor neurons. To identify and subsequently quantify the synapses on motor neurons, the two structures (synapses and motor neurons) need to be in different fluorochromes and we opted to investigate the proprioceptive synapses originating from the TA muscle. To label proprioceptive fibers and synapses originating from the TA muscle only, we injected PV::FlpO^+/+^;Ai65^+/+^ mice at birth (P0) with the CAV2-Cre virus in the TA muscle (Fig. S2 I). CRE expression would ensure labelling of proprioceptive fibers and their synapses since parvalbumin (PV) is expressed in proprioceptive neurons. To differentiate between sensory fibers (axons) and synapses, we performed immunohistochemistry against VGluT1, a marker for synapses but not axons/fibers. VGluT1^+^ synapses that contact the somata and dendrites of motor neurons are of proprioceptive origin only. To label TA motor neurons only with a different color/fluorochrome, we co-injected the TA muscle with AAV6-GFP. In this manner, the AAV6-GFP would mark motor neurons only (via retrograde transport) and would not label proprioceptive fibers nor their synapses, as previously demonstrated (Balaskas et al., 2019). We found that TA motor neurons in all genotypes (WT, C3^−/−^, and CD47^−/−^) received appropriate proprioceptive synapses (Fig. S2 J) and quantification revealed that ∼55% of the VGluT1^+^ proprioceptive synapses on the motor neuron soma were appropriate synapses (i.e., originating from the TA muscle; Fig. S2 K). However, there was no statistical difference among any of the three experimental groups (Fig. S2 K). These results indicate that the supernumerary synapses observed in the mutant mice are inappropriate synapses, while appropriate synapses are not affected in C3^−/−^ and CD47^−/−^ mice.

Taken together, these results demonstrate that excessive synapses between proprioceptors and antagonistic motor neurons are formed during early development, resulting in miswired sensory-motor circuits, and this miswiring is not attributed to the location of motor neurons.

### Inappropriate sensory synapses are functional, resulting in improper muscle contraction

**Figure 5.**
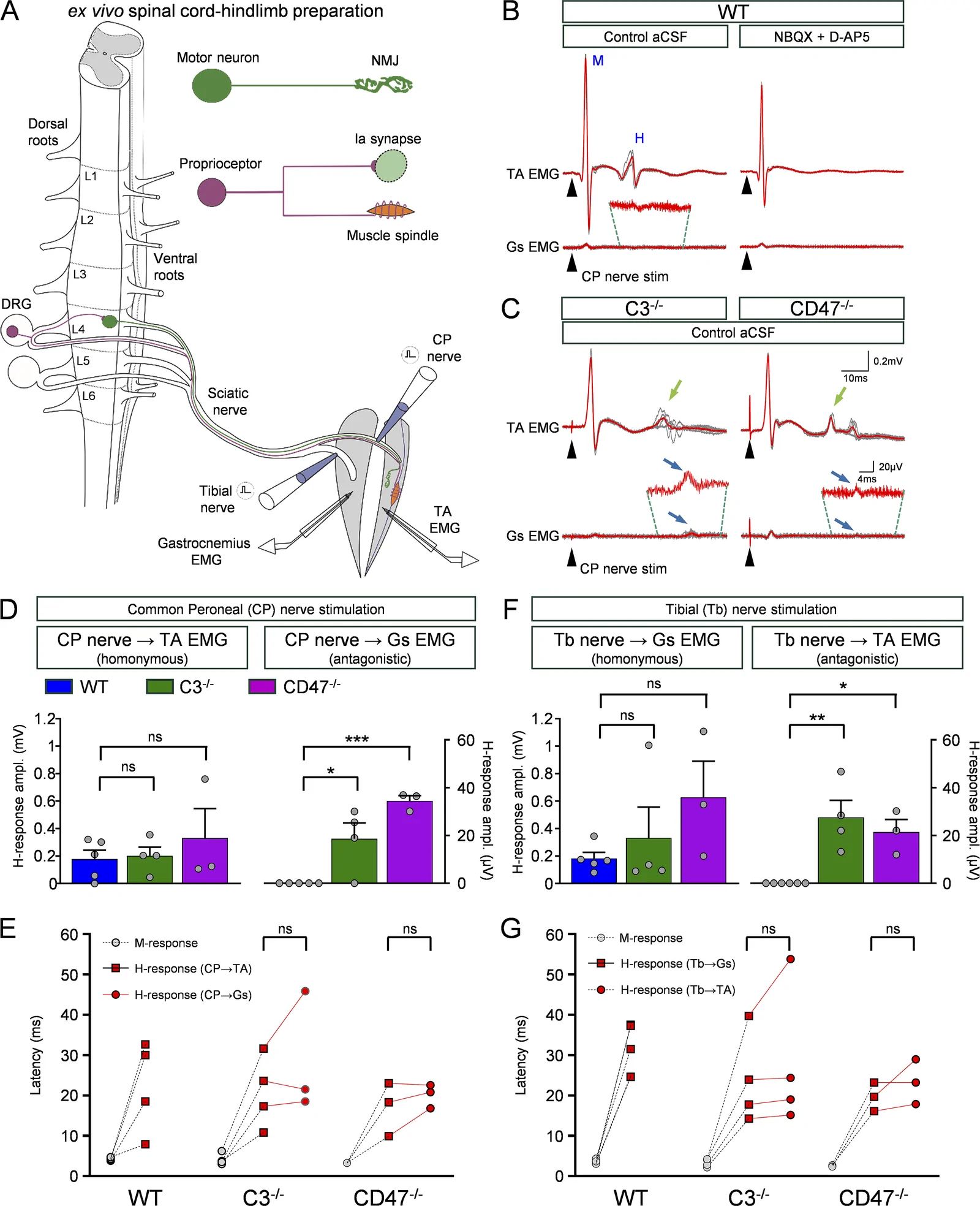
**Inappropriate proprioceptive sensory synapses on motor neurons induce responses in antagonistic muscles. (A)** Schematic of the ex vivo spinal cord–hindlimb preparation. Suction electrodes were placed on the CP and Tb nerves to perform en passant stimulation. Bipolar concentric needle electrodes recorded EMG activity from the TA and Gs muscles. A motor neuron and a neuromuscular junction (NMJ) are shown in green. A proprioceptor with its muscle spindle and the Ia central synapse on motor neurons are shown in purple. **(B)** Simultaneous EMG recordings from TA and Gs muscles following CP nerve stimulation in a WT spinal cord under control aCSF solution and after exposure to NBQX (20 μM) and D-AP5 (50 μM). Red traces show the average of five trials (grey). Black arrowhead denotes a stimulus artifact. **(C)** Traces from C3^−/−^ (left) and CD47^−/−^ (right) mice in control aCSF. Green arrows point to homonymous muscle H-reflex, while blue arrows point to inappropriate H-responses from the antagonistic Gs muscle following CP nerve stimulation. **(D)** Amplitude of H-reflex induced by CP nerve stimulation in the homonymous TA muscle (left; CP nerve → TA EMG) and the H-response from the antagonistic Gs muscle (right; CP nerve → Gs EMG). **(E)** Latency measurements of M-responses evoked by CP nerve stimulation in the TA muscle (grey circles), as well as H-reflex in the TA muscle following CP nerve stimulation (red squares), and antagonistic H-responses in the Gs muscle following CP nerve stimulation (red circles). **(F)** Amplitude of H-reflex induced by Tb nerve stimulation in the homonymous Gs muscle (left; Tb nerve → Gs EMG) and the H-response from the antagonistic TA muscle (right; Tb nerve → TA EMG). Each data point corresponds to a single animal (WT: *N* = 5; C3^−/−^: *N* = 4; CD47^−/−^: *N* = 3 mice). Significance: *P < 0.05, **P < 0.01, ***P < 0.001; one-way ANOVA with multiple comparisons using Bonferroni’s test. ns: no significance. **(G)** Latency measurements of M-responses evoked by Tb nerve stimulation in the Gs muscle (grey circles), as well as H-reflex in the Gs muscle following Tb nerve stimulation (red squares), and antagonistic H-responses in the TA muscle following Tb nerve stimulation (red circles). One-way ANOVA with multiple comparisons using Bonferroni’s test; ns: no significance.

We next investigated whether the inappropriate synapses are functional by performing H-reflex (or Hoffman reflex) experiments using electromyograms (EMG) in the ex vivo spinal cord–hindlimb preparation (Mentis et al., 2005; Bikoff et al., 2016) at P5 (Fig. 5 A). The H-reflex is the reaction in muscles following electrical stimulation of proprioceptive sensory fibers within their innervating nerves through the spinal cord (Fig. 5 A) (Chen et al., 2003; Kudo and Yamada 1985). The M-response is the muscle response following direct stimulation of motor neuron axons within the stimulating nerve. In WT mice, stimulation of the common peroneal (CP) nerve resulted in a robust M-response and an H-response (or H-reflex) in the TA muscle (Fig. 5 B). In addition, simultaneous recordings from the antagonistic muscle Gs did not result in any responses, as expected in healthy (WT) control mice (Fig. 5 B). We confirmed that the neurotransmitter responsible for the production of the H-reflex was glutamate, since exposure to the NMDA receptor blocker D-AP5 (50 μM) and the AMPA/kainate receptor blocker NBQX (20 μM) abolished the response, leaving relatively unaffected the M-response (Fig. 5 B, right side traces). In C3^−/−^ and CD47^−/−^ mutant mice, however, a response with a latency similar (or slightly delayed) to the H-reflex was detected in the antagonistic Gs muscle following CP nerve stimulation (Fig. 5 C; and insets). The amplitude of the H-reflex induced in the homonymous muscle (CP nerve stimulation to TA muscle) did not reveal any significant differences between the three experimental groups (Fig. 5 D in CP→TA). In contrast, we observed an inappropriate response in the antagonistic Gs muscle, with similar latency to that of the H-reflex observed in the homonymous TA muscle (Fig. 5, C and D in CP→Gs, and Fig. 5 E) only in C3^−/−^ and CD47^−/−^ mice. This result suggests that inappropriate synapses derived from TA muscle afferents are sufficient to activate Gs motor neurons, resulting in improper Gs muscle contraction (Fig. 5 C, blue arrows). Similar results were obtained when we tested the stimulation of the tibial (Tb) nerve recorded in the TA muscle (Fig. 5, F and G).

**Figure S3.**
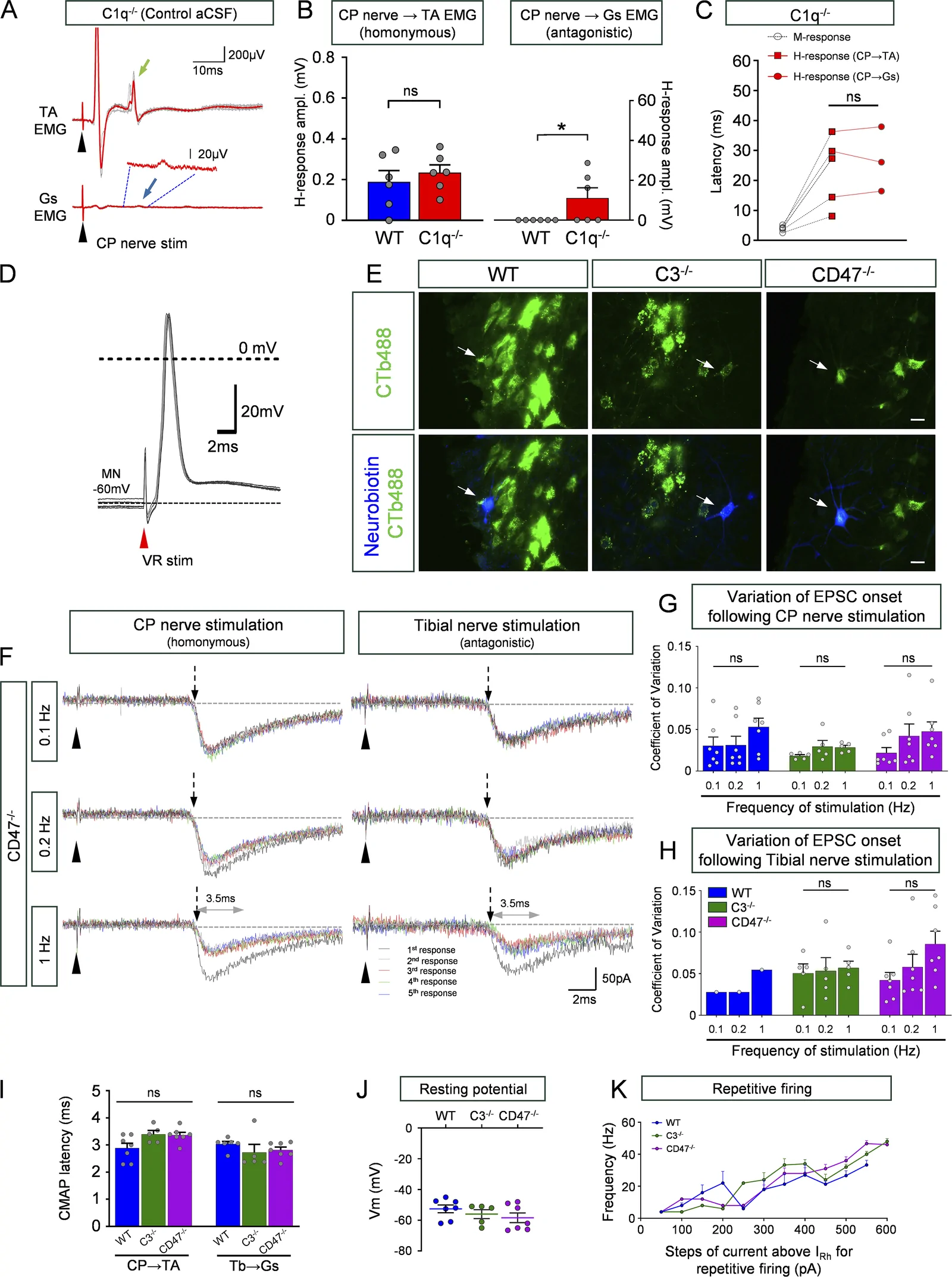
**H-responses evoked by antagonistic nerve stimulation in C1q** ^**−/−**^ **mice and intrinsic properties and morphological identification of TA motor neurons in WT, C3** ^**−/−**^ **and CD47** ^**−/−**^ **mice. (A)** EMG responses from the TA and Gs muscles in response to CP nerve stimulation in a C1q^−/−^ mouse. Red traces depict the average of five responses (shown in grey) acquired at 0.1 Hz. Black arrowheads indicate stimulation artifact. Green arrow indicates the H-reflex. Blue arrow indicates the inappropriate H-response in the antagonistic muscle (magnified in the inset). **(B)** Amplitude measurements of H-response following CP nerve stimulation in the TA muscle (CP nerve → TA EMG) and in the antagonistic Gs muscle (CP nerve → Gs muscle) in WT and C1q^−/−^ mice. WT: *N* = 6 mice, C1q^−/−^: *N* = 6. Significance: *P < 0.05; unpaired *t* test; ns: no significance. **(C)** Latency of responses following CP nerve stimulation for M-response (empty circles), for H-reflex in the TA muscle (red squares), and for H-response in the antagonistic Gs muscle (red circles). In one mouse, no response was evident in the Gs muscle. ns: no significance, unpaired *t* test. **(D)** Superimposed antidromic action potentials in TA motor neuron following stimulation of the L4 ventral root. Red arrowhead indicates stimulation artifact. **(E)** Single optical plane confocal images of visualized Neurobiotin-filled motor neurons confirming their identity as of those innervating the TA muscle. Arrows show colocalization between Neurobiotin (blue) and CTb488 (green) (scale bar: 20 μm). **(F)** Jitter test of latency confirms monosynapticity of EPSCs. Example of superimposed EPSCs evoked at 0.1 Hz, 0.2 Hz, and 1 Hz. Vertical dotted line with arrows indicates time-locked onset of EPSC at different frequencies for either homonymous (CP nerve) or antagonistic (Tb nerve) stimulation. Recordings from a TA motor neuron in a CD47^−/−^ mouse at P5. Black arrowheads indicate stimulus artifact. **(G and H)** Coefficient of variation for the latency of the EPSC onset in TA motor neurons following: (G) CP (homonymous) nerve stimulation and (H) Tb (antagonistic) nerve stimulation. ns: no significance, One-way ANOVA, Tukey’s post hoc test. **(I)** CMAP latency in WT, C3^−/−^ and CD47^−/−^ mice, following stimulation of CP nerve while recording in the TA muscle, or stimulation of the Tb nerve while recording from the Gs muscle. **(J)** Resting membrane potential of recorded TA motor neurons in WT, C3^−/−^ and CD47^−/−^ mice. Only one motor neuron was recorded per mouse. WT: *N* = 7 mice; C3^−/−^: *N* = 5; CD47^−/−^: *N* = 7. **(K)** Firing frequency-to-current relationship in TA motor neurons from WT, C3^−/−^ and CD47^−/−^ mice. ns: no significance. One-way ANOVA, post hoc Bonferroni’s test. CAMP, compound muscle action potential.

We next investigated the hypothesis that if the classical complement cascade is involved in the refinement of inappropriate synapses, then C1q^−/−^ mice are expected to exhibit similar inappropriate responses to C3^−/−^ mice. To this end, our experiments using C1q^−/−^ mice resulted in inappropriate H-reflex responses in the Gs muscle following stimulation of the antagonistic CP nerve (Fig. S3, A–C), thus validating our hypothesis. These results demonstrate that both C3 and CD47 proteins participate in the pruning of supernumerary synapses, formed during embryonic and early postnatal development.

**Figure 6.**
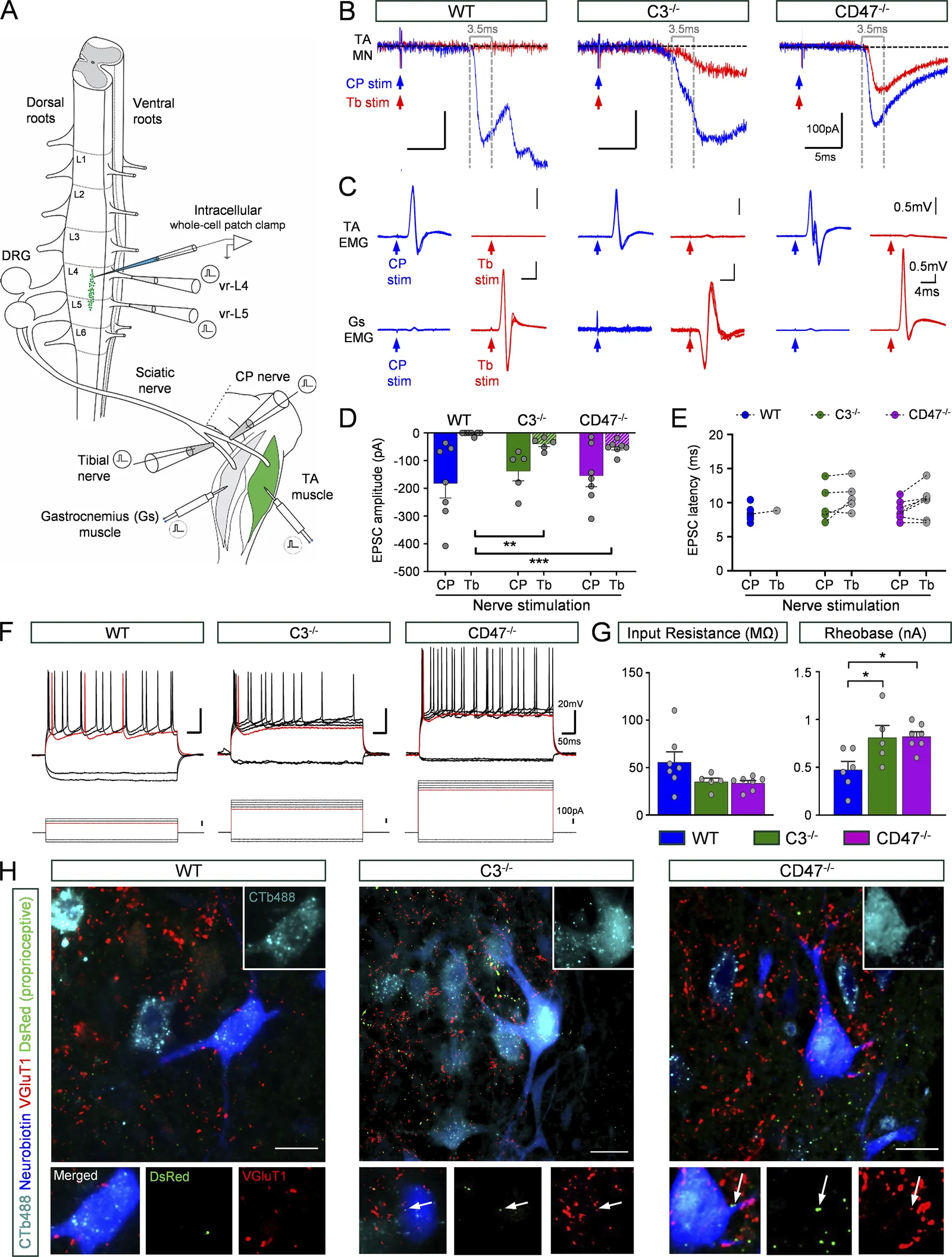
**Inappropriate synapses evoke monosynaptic EPSCs. (A)** Schematic of the ex vivo spinal cord–hindlimb preparation utilized for whole-cell patch-clamp from TA motor neurons. Bipolar concentric needle electrodes were placed in the TA and Gs muscles to record EMG. Suction electrodes were placed in the CP and Tb nerves for stimulation en passant*.* The L4 and L5 ventral roots were cut and placed in suction electrodes. Intracellular electrode contained Neurobiotin. At birth, the TA muscle was injected with CTb-488, while the Gs muscle was injected with CAV2^CRE^ virus in PV::FlpO;Ai65-TdTomato mice. **(B)** Whole-cell responses from TA motor neurons following stimulation of the homonymous CP nerve (blue) or the antagonistic Tb nerve (red) in WT, C3^−/−^, and CD47^−/−^ mice at P4–6. The EPSC response is the mean of five responses (evoked at 0.1 Hz). Dotted lines depict the duration of 3.5 ms, which was used as the criterion for monosynaptic duration. **(C)** EMG responses corresponding to the experimental groups as shown in B above. The TA (top traces) and Gs EMG responses (bottom traces) were acquired concurrently following stimulation of either the CP nerve (blue) or the Tb nerve (red). Superimposed traces shown are five responses evoked at 0.1 Hz. **(D)** Amplitude of EPSC evoked in TA motor neurons following CP (plain bars) or Tb (hatched bars) nerve stimulation in WT (blue), C3^−/−^ (green), and CD47^−/−^ (purple) mice (WT: *n* = 7 motor neurons; C3^−/−^: *n* = 5; CD47^−/−^: *n* = 7). One motor neuron per mouse was recorded. **(E)** Latency of EPSC for experimental groups shown in D. Note that in WT mice, only one TA motor neuron exhibited a small EPSC following Tb nerve stimulation. **(F)** Voltage responses in TA motor neurons following steps of current injection (in patch-clamp experiments) in WT, C3^−/−^, and CD47^−/−^ mice (as shown in B). **(G)** Input resistance (left) and rheobase (right) of TA motor neurons in the three experimental groups. Significance: *P < 0.05, **P < 0.01, ***P < 0.001; one-way ANOVA multiple comparisons with Bonferroni’s test. **(H)** Confocal images of recorded, filled, and subsequently visualized TA motor neurons from the three experimental groups: PV::FlpO;Ai65 (WT), PV::FlpO;Ai65;C3^−/−^ (C3^−/−^), and PV::FlpO;Ai65;CD47^−/−^ (CD47^−/−^) mice. Images correspond to TA-recorded motor neurons for each mouse line from experiments shown in B, C, and F. Quadruple immunostaining against VGluT1 (red), DsRed (green), Neurobiotin (blue), and injected CTb488 (magenta). Images are Z-stack projections of single optical planes totaling 3.5 μm in the Z-axis. Inserts are single optical planes, corresponding to higher magnification of the recorded motor neuron soma (top; CTb); white arrow indicates inappropriate synapses on the soma of the TA motor neuron in C3^−/−^ and CD47^−/−^ mice only (bottom images). Scale bar: 20 μm.

To exclude the possibility that the inappropriate responses in the antagonistic muscles were F-waves (Fisher 2007), we stimulated the nerved repeatedly at low frequencies (∼0.1 Hz) and found that the responses were time locked. In contrast, F-waves were not time locked (data not shown). Furthermore, the responses appeared earlier than the typical F-wave responses, which were variable in amplitude and latency (data not shown). The latency of the inappropriately evoked EMG response in the Gs muscle after CP nerve stimulation varied considerably (range: −1 to +15 ms; Fig. 5, E–G), raising the possibility that inappropriate proprioceptive synapses might not be synapsing directly on motor neurons. To resolve this, we performed whole-cell patch-clamp recordings using the ex vivo spinal cord–hindlimb preparation to record intracellular responses from TA motor neurons following selective peripheral nerve stimulation (Fig. 6 A). Ventral roots L4 and L5 were cut to avoid contamination of synaptic currents by the antidromic action potential induced by the homonymous (CP) nerve stimulation. Both L4 and L5 ventral roots were placed in suction electrodes, so the patched neuron could be identified as a motor neuron by the presence of an antidromic action potential (Fig. S3 D). TA motor neurons were visually targeted for whole-cell intracellular recordings by the presence of CTb-488, which was previously injected into the TA muscle at P0 (Fig. S3 E). Excitatory postsynaptic currents (EPSCs) induced by either CP nerve (homonymous) or Tb nerve (antagonistic) stimulation were compared in WT, C3^−/−^, and CD47^−/−^ mice. To assess if the responses were monosynaptic in nature, we analyzed the latency of the EPSCs at different stimulation frequencies (0.1, 0.2, and 1 Hz) and found that the coefficient of variation (jitter test) was not significantly different (Fig. S3, F–H), demonstrating monosynapticity. Our criterion for the duration of monosynaptic transmission in our neonatal ex vivo spinal cord was 3.5 ms, as we previously reported (Mendelsohn et al., 2015).

Stimulation of the homonymous nerve (CP) resulted in robust monosynaptic EPSCs in TA motor neurons in all experimental groups (blue traces in Fig. 6 B), with a similar amplitude on average (Fig. 6 D). In striking contrast, stimulation of the antagonistic nerve (Tb) resulted in monosynaptic EPSCs only in C3^−/−^ and CD47^−/−^ mice, but not in their WT counterparts (red traces in Fig. 6 B). This indicates that responses evoked 3.5 ms after the onset of the EPSC must be polysynaptic in nature. Importantly, the resultant EPSC latency revealed no significant differences for either CP or Tb nerve stimulation (Fig. 6 E). Appropriate stimulation of each peripheral nerve was verified by EMG responses recorded in both TA and Gs muscles simultaneously (Fig. 6 C). The compound muscle action potential latency was not significantly different in the homonymous muscle (CP→TA or Tb→Gs) in all three groups (Fig. S3 I). These data demonstrate that inappropriate proprioceptive synapses result in monosynaptically induced EPSCs in C3^−/−^ and CD47^−/−^ mice.

We next investigated the effects of inappropriate synapses on the active and passive membrane characteristics of TA motor neurons. We found that resting membrane potential in all TA motor neurons was not statistically different across the three groups (Fig. S3 J). The input resistance revealed a trend towards a reduction, while the rheobase current was significantly higher in both mutant mice compared with WT controls (Fig. 6, F and G). The moderate increase in rheobase (Fig. 6 G) may underlie the reason for the trend in the increased repetitive firing ability in C3^−/−^ and CD47^−/−^ motor neurons (Fig. S3 K).

To reveal the site of the inappropriate synapses on recorded TA motor neurons, we labelled motor neurons with Neurobiotin (added in the intracellular solution), and visually revealed by immunohistochemistry post hoc (blue in Fig. 6 H and Fig. S3 E). Proprioceptive fibers originating in Gs muscles were labelled using our strategy explained previously (Fig. 3 A), in which CAV2^CRE^ was injected in Gs muscle. TA motor neurons were marked by CTb-488 (cyan in Fig. 6 H and green in Fig. S3 E) injected into the TA muscle. In this assay, we found that inappropriate synapses were located on the soma and proximal dendrites of TA motor neurons, only in C3^−/−^ and CD47^−/−^ mice (white arrows in Fig. 6 H), but importantly, not in WT mice. Taken together, these results demonstrate that inappropriate synapses on TA motor neurons are functional and alter their intrinsic properties, likely impacting muscle contraction. In addition, they suggest that inappropriate synapses are eliminated via activation of the classical complement cascade and CD47-mediated mechanisms during early development.

### Expression of CD47 and its receptor SIRPα in the spinal cord

**Figure 7.**
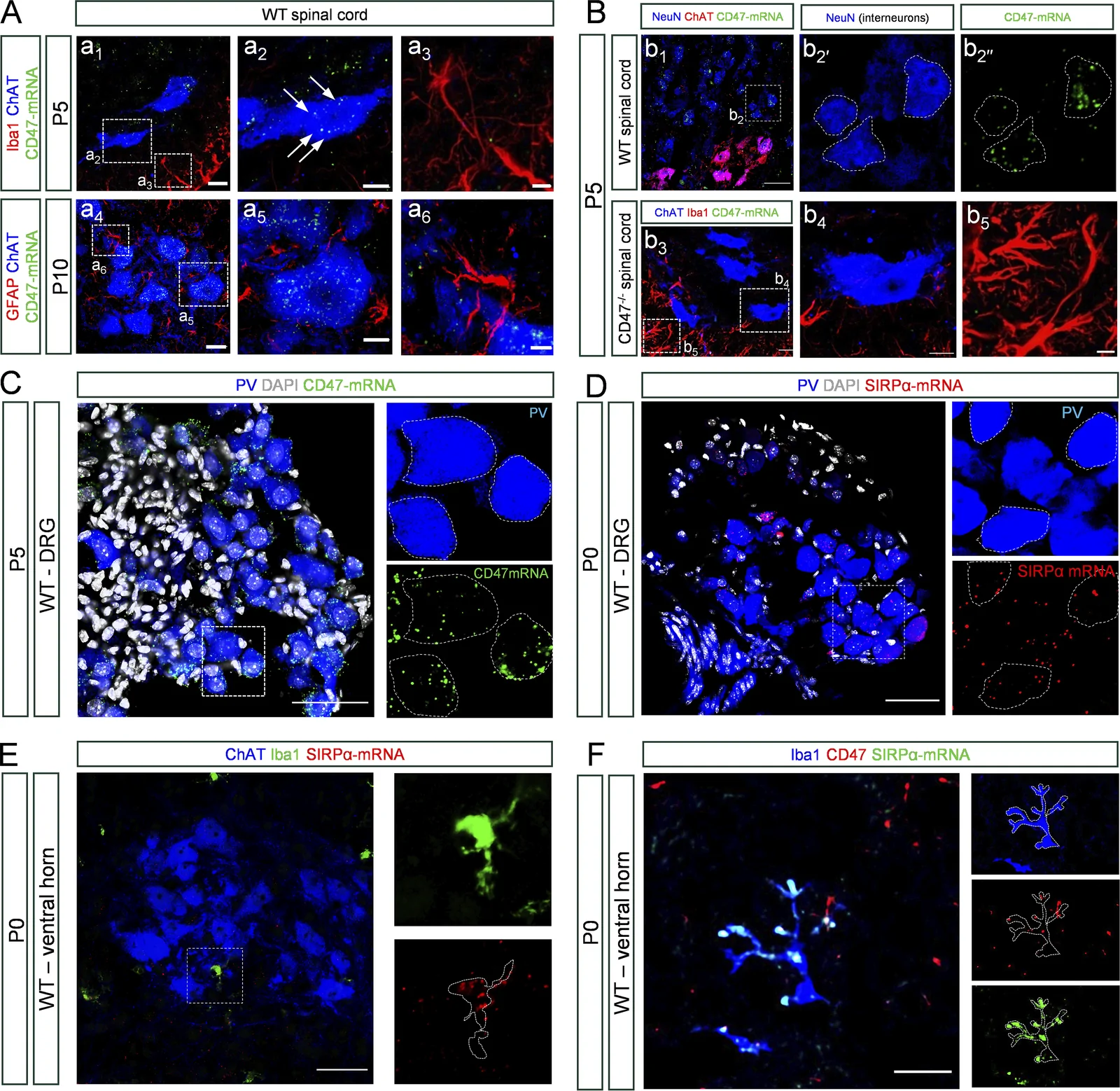
**Neurons express CD47, while microglia express SRIPα. (A)** Z-stack confocal images (total distance in Z-axis: 3.5 μm) from a P5 L4 spinal cord showing motor neurons (ChAT; blue), microglia (Iba1; red), and CD47-mRNA (RNAscope, in green) at P5 (top row). At P10 (bottom row), images show motor neurons (ChAT; blue), astrocytes (GFAP, red), and CD47-mRNA (green). Scale bar in a_1_ and a_4_: 10 μm. Middle and right columns correspond to higher magnification images marked by dotted boxes in a_1_ and a_4_, respectively. White arrows indicate the CD47-mRNA puncta. Scale bar in a_2_, a_3_, a_5_, and a_6_: 5 μm. **(B)** Confocal images of CD47-mRNA puncta in interneurons, labelled by NeuN (blue), motor neurons (ChAT; red), and CD47-mRNA (green). Scale bar in b_1_: 50 μm. b_2' _image shows NeuN signal from dotted box in b_1_. b_2''_ shows CD47 mRNA signal for the same area as b_2'_. Dotted lines mark the perimeter of the neuronal soma. Bottom row of images shows validation of CD47-mRNA specificity using an RNAscope assay in a CD47^−/−^ mouse, with immunohistochemistry against ChAT (b_4_) and Iba1 (b_5_). Scale bar in b_3_, b_4_, b_5_: 10 μm. **(C)** Confocal images of dorsal root ganglia (DRG) showing immunoreactivity against PV (blue), DAPI signal (white), and RNAscope probe against CD47-mRNA (green). Scale bar: 50 μm. **(D)** Confocal image of PV (blue), DAPI (white), and SIRPa-mRNA (red). Scale bar: 50 μm. **(E)** Confocal image showing motor neurons (ChAT, blue), microglia (Iba1, green), and SIRPα-mRNA (red). Scale bar: 50 μm. Insets on the right, show higher magnification of Iba1 and SIRPα-mRNA in dotted box area. **(F)** Single optical plane of confocal image showing immunoreactivity for microglia (Iba1, blue) and CD47 (red) and RNAscope signal against SIRPα-mRNA (green). Scale bar: 5 μm.

Since our results show that that CD47 promotes synaptic pruning, an unexpected finding, we next sought to identify the source of CD47 in the spinal cord. We performed RNAscope experiments combined with immunohistochemistry assays in neonatal animals (P0–P10). We found that CD47 was expressed only in neurons, including motor neurons (identified by ChAT immunoreactivity) (Fig. 7 A), spinal interneurons in the intermediate grey matter (identified by NeuN antibodies in the ventral horn) (Fig. 7 B), and proprioceptive neurons (identified by PV immunoreactivity) (Fig. 7 C). This is in partial agreement with a recent report in which CD47 was only expressed in postsynaptic, but not presynaptic, sites in the brain (Jiang et al., 2022), unlike our study, where we observe CD47 in both presynaptic (proprioceptor) and postsynaptic (motor neuron) sites. CD47 was not expressed in either microglia (identified by Iba1 immunoreactivity) or astrocytes (identified by GFAP immunoreactivity; Fig. 7, A and B). Importantly, the CD47 RNAscope probe was validated in the CD47^−/−^ spinal cord, in which no signal was detected (Fig. 7 B; bottom row images). SIRPα —the CD47 receptor—was expressed exclusively in proprioceptors (Fig. 7 D) and microglia (Fig. 7, E and F), but not in motor neurons (Fig. 7 E).

### CD47 and C3 tag proprioceptive synapses on motor neurons

We next investigated the potential molecular mechanism involved in synaptic pruning and regulated by CD47 activity. CD47 has been described as the main molecule involved in synapse protection in the brain. However, in complete contrast, the results of our study demonstrate that CD47 participates in synaptic elimination, an unanticipated function for CD47. To delve deeper into this function of CD47, we investigated whether CD47 expression was involved in synaptic tagging and engulfment by microglial cells in the developing spinal cord.

**Figure 8.**
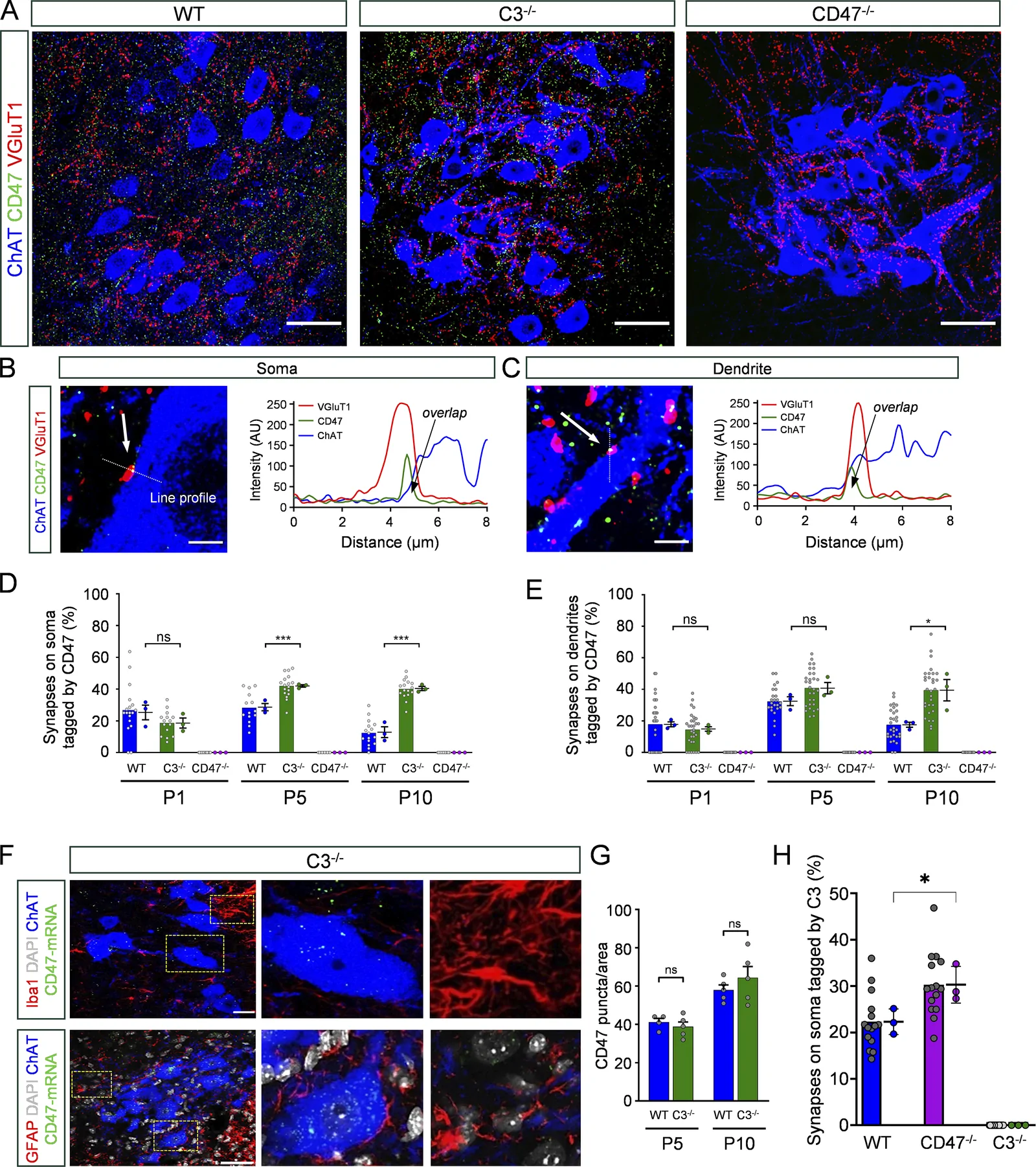
**CD47 tags proprioceptive synapses in spinal sensory-motor circuits. (A)** Single-plane confocal images of VGluT1 (red), CD47 (green), and ChAT (blue) immunoreactivity in WT, C3^−/−^, and CD47^−/−^ mice at P5. Scale bar: 50 μm. **(B)** VGluT1^+^ synapse (red) opposed to a motor neuron soma (ChAT, blue) is tagged by CD47 (green). Graphs show that the CD47 signal intensity overlaps with that of VGluT1, indicated by the line profile (arrow, dotted line), for the three antibodies. Scale bar: 5 μm. **(C)** Similar to B but for a synapse on a motor neuron dendrite. Scale bar: 5 μm. **(D)** Percentage of somatic synapses tagged by CD47 at P1, P5, and P10. **(E)** Percentage of dendritic synapses tagged by CD47. Each dot (in grey) corresponds to one motor neuron. Colored data points show average values from a single mouse (*N* = 3 mice/group). Number of motor neurons analyzed from *N* = 3 mice (WT: P1, *n* = 30; P5, *n* = 24; P10, *n* = 30) (C3^−/−^: P1, *n* = 30; P5, *n* = 30; P10, *n* = 30) (CD47^−/−^: P1, *n* = 30; P5, *n* = 20; P10, *n* = 30). **(F)** Confocal images showing CD47-mRNA (RNAscope, in green) and immunoreactivity signals with ChAT (blue), Iba1 (red, top row), GFAP (red, bottom row) and DAPI (white) in C3^−/−^ mice at P5. Dotted boxes indicate areas of higher magnification shown on the right. Scale bar: 20 μm. **(G)** Quantification of CD47 puncta per soma in WT (P5, *N* = 4 mice; P10, *N* = 5) and C3^−/−^ (P5, *N* = 5; P10, *N* = 5). **(H)** Percentage of somatic synapses tagged by C3 at P5. Number of motor neurons analyzed from *N* = 3 mice per genotype (WT: *n* = 15, CD47^−/−^: *n* = 15, C3^−/−^: *n* = 15). Statistical comparison was performed across averages from mice. Significance: ns = no significance, *P < 0.01, ***P < 0.001; one-way ANOVA with multiple comparisons using Bonferroni’s test; ns: no significance.

**Figure S4.**
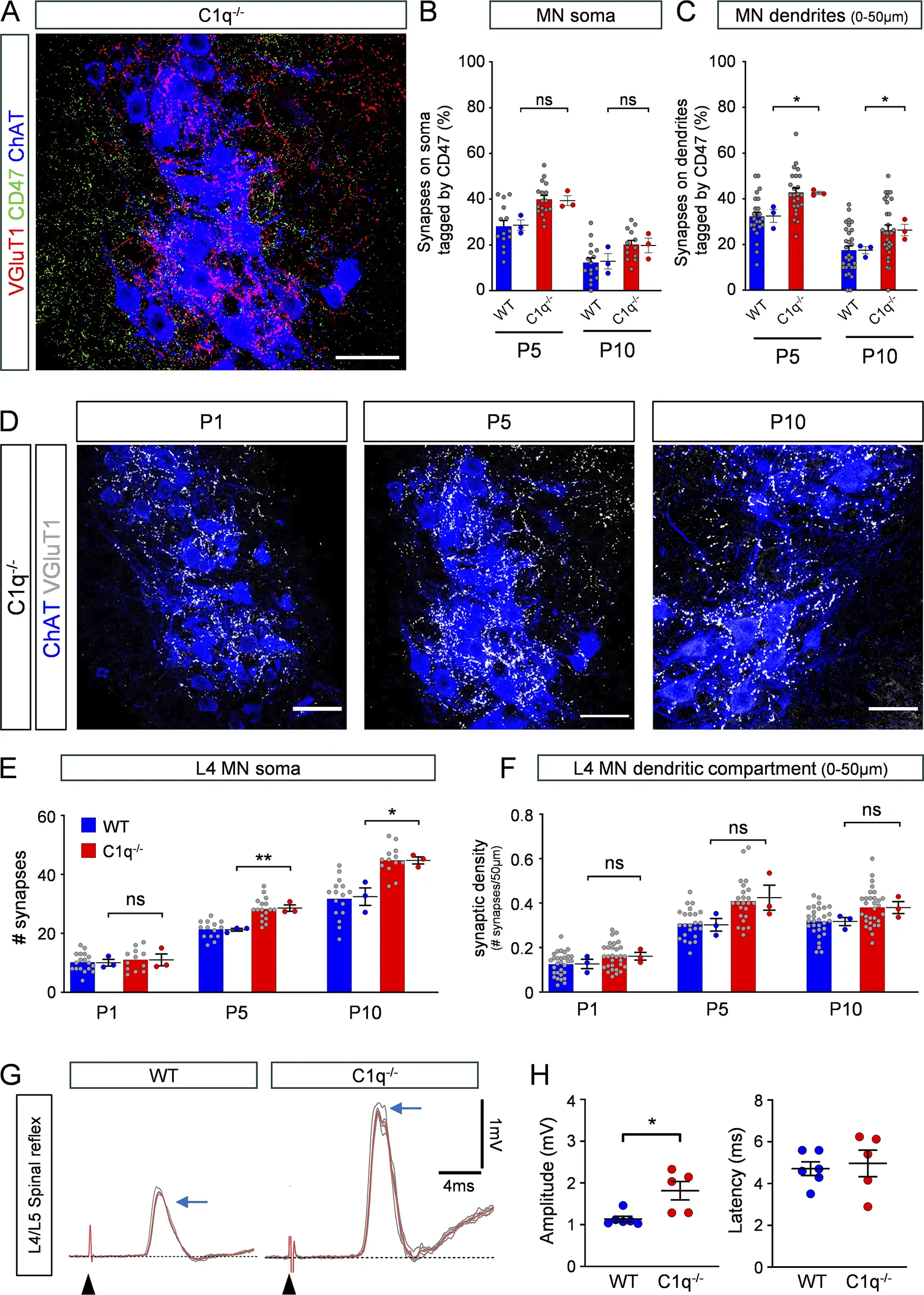
**Effects of C1q genetic deletion on the sensory-motor circuit. (A)** Single-plane confocal image of VGluT1 (red), CD47 (green) and ChAT (blue) immunoreactivity in a C1q^−/−^ mouse at P5. Scale bar: 50 μm. **(B)** Percentage of somatic synapses tagged by CD47 in WT (blue) and C1q^−/−^ (red) mice at P5 and P10 (*N* = 3 mice/group; colored points in the graph). Grey data points (and bars) represent the number of synapses analyzed in: WT: *n* = 14 at P5, *n* = 16 at P10; C1q^−/−^: *n* = 17 at P5, *n* = 13 at P10 for *N* = 3 mice/group. Colored points represent average values per mouse. **(C)** Percentage of dendritic synapses tagged by CD47. Number of primary dendrites (0–50 μm from the soma); WT: *n* = 24 at P5, *n* = 30 at P10; C1q^−/−^: *n* = 24 at P5, *n* = 30 at P10 (grey points) for *N* = 3 mice/group (colored points represent average values per mouse). Significance: *P < 0.05; One-way ANOVA multiple comparisons with Bonferroni’s test. **(D)** Confocal images of L4/5 spinal cord ventral horns from C1q^−/−^ mice at P1, P5, and P10 showing immunoreactivity against ChAT (blue) and VGlut1 (white). Scale bar: 50 μm. Image shown in A is the same image as shown in P5 (middle image) with the exception that VGluT1 synapses appear in white for clarity and comparison with images at P1 and P10. **(E)** Number of VGluT1 synapses per motor neuron soma at P1, P5, and P10 mice (*N* = 3 mice/group; colored points in the graph) in WT and C1q^−/−^ mice. Number of motor neuron somata analyzed, WT: *n* = 18 at P1, *n* = 14 at P5, *n* = 16 at P10; C1q^−/−^: *n* = 12 at P1, *n* = 17 at P5, *n* = 13 at P10. *P < 0.05, **P < 0.01, One-way ANOVA, Tukey’s post hoc test. **(F)** Synaptic density (# synapses/50 μm dendrite) for proximal dendrites of motor neurons (*N* = 3 mice/group; colored points in the graph). Number of dendrites analyzed, WT: *n* = 30 at P1, *n* = 24 at P5, *n* = 30 at P10; C1q^−/−^: *n* = 30 at P1, *n* = 24 at P5, *n* = 30 at P10. ns: no significance. **(G)** Electrical responses from ventral roots following stimulation of the L4 (or L5) dorsal root in WT and C1q^−/−^ mice. Red traces show the average of first five responses (grey) acquired at 0.1 Hz. Arrowhead indicates stimulus artifact. The dotted line indicates baseline. Horizontal blue arrows indicate peak amplitude of the averaged response. **(H)** Amplitude and latency of spinal reflexes at P5 in WT (blue, *N* = 6 mice) and C1q^−/−^ (red, *N* = 5) mice. Significance: *P < 0.05, Unpaired *t* test.

We performed immunohistochemistry experiments in which motor neurons (ChAT^+^) and their proprioceptive synapses (VGluT1^+^) were investigated for their potential association with CD47 at P1, P5, and P10 in WT, C3^−/−^, and CD47^−/−^ mice using a validated anti-CD47 antibody (Fig. 8 A). CD47 was considered as tagging VGluT1^+^ synapses (apposed on either soma or dendrites of motor neurons) when there was an overlap of their corresponding immunoreactive optical signals (white arrows and graphs in Fig. 8, B and C). The number of CD47-tagged VGluT1^+^ synapses was significantly increased at P5 and P10 in C3^−/−^ mice, but not at P1 (Fig. 8, A, D, and E) compared with WT mice. CD47 antibody specificity was validated in CD47^−/−^ mice (Fig. 8, A, D, and E). CD47’s increased synaptic tagging was also evident in C1q^−/−^ proprioceptive (VGluT1^+^) synapses on motor neurons, both on their soma and dendrites from P5 until P10 (Fig. S4, A–C). To this end, genetic knockout of C1q ubiquitously resulted in a higher incidence of proprioceptive synapses on motor neurons from P5 onward (Fig. S4, D–F), in agreement with our previous report that the classical complement cascade is involved in synaptic elimination (Vukojicic et al., 2019). Furthermore, the amplitude of the monosynaptic responses in C1q^−/−^ mice was also significantly higher compared to age-matched WT controls at P5 (Fig. S4, G and H).

Since CD47-tagging was higher in C3^−/−^ and C1q^−/−^ mice, we investigated whether CD47 might be upregulated in motor neurons. However, we observed no differences in CD47 mRNA puncta in TA motor neurons from C3^−/−^ mice (Fig. 8, F and G), suggesting that CD47 tagging was unlikely to originate from motor neurons. In addition, there was no CD47 mRNA expression in either microglia or astrocytes in C3^−/−^ mice, similar to their WT counterparts (Fig. 8 F).

We also quantified the percentage of VGluT1 synapses (on the motor neuron soma) tagged by C3 in WT, CD47^−/−^, and C3^−/−^ (as controls) mice. Intriguingly, we found a significant increase in the percentage of C3-tagged synapses in CD47^−/−^ mice compared with WT counterparts (Fig. 8 H). These results indicate that CD47 tagging of proprioceptive synapses is independent of the classical complement cascade and proprioceptive synapses may be removed, by being tagged by either C3 or CD47. Thus, synaptic elimination could result from a cooperation between activation of C3 and CD47-mediated molecular mechanisms, although we cannot exclude that additional mechanisms—including C1q tagging—may be involved in synaptic tagging and subsequent synaptic elimination.

### VGluT1 synaptic remnants tagged with either CD47 or C3 are engulfed by microglia

**Figure 9.**
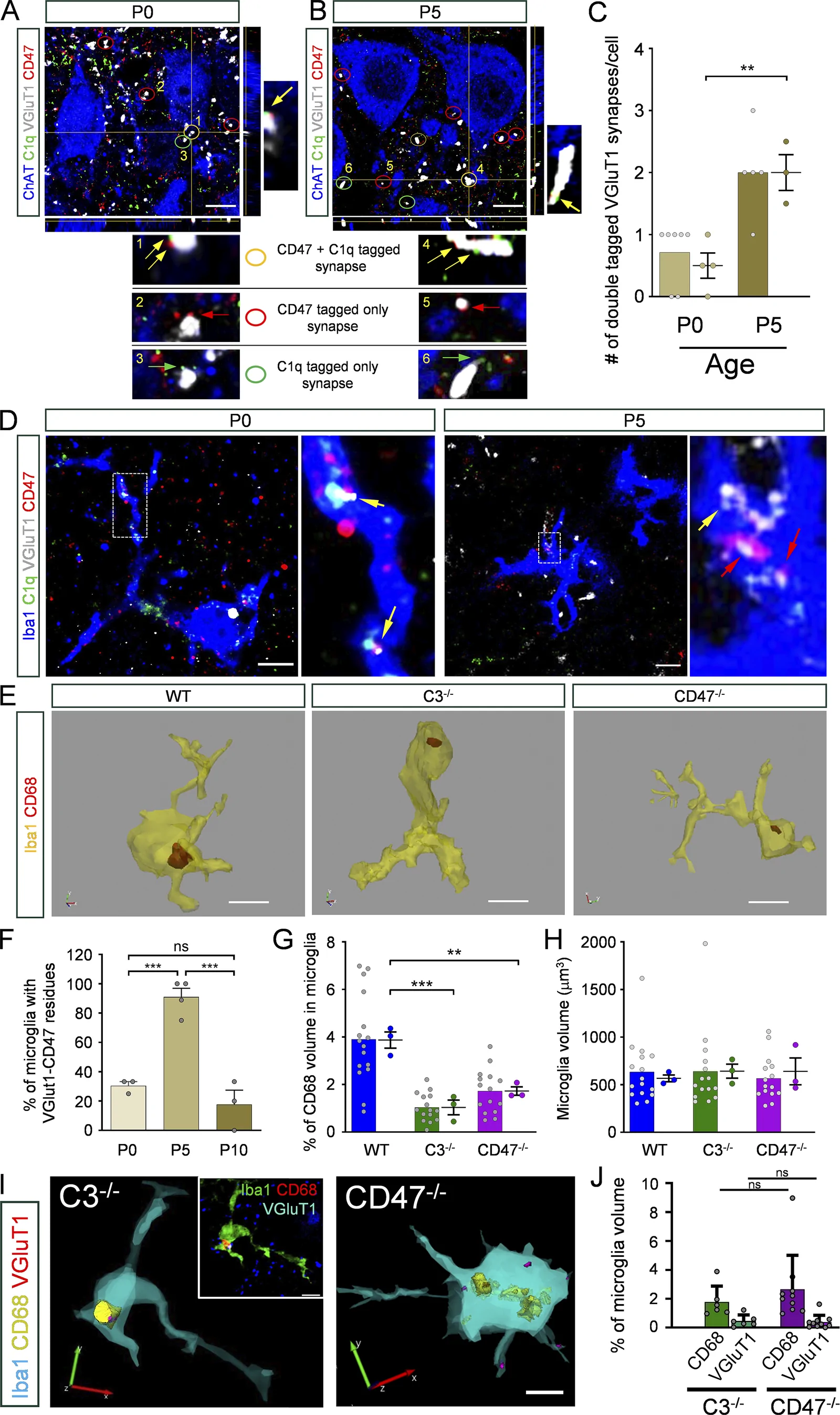
**Microglia engulf synapses tagged by C1q, CD47, or combined. (A and B)** Orthogonal view of images immunoreactive against C1q (green), CD47 (red), VGlut1 (white), and ChAT (blue) at P0 (A) and P5 (B). Circles indicate instances where a VGlut1 synapse is tagged by CD47 only (red circle), C1q only (green circle), or combined by both CD47 and C1q (yellow circle). Inserts at the bottom represent higher magnifications. Yellow arrows indicate double tagging of VGluT1 synapses by CD47 and C1q. Red arrows indicate tagging by CD47 only. Green arrows indicate tagging by C1q only. Scale bar in A and B: 10 μm. **(C)** Quantification of VGluT1 synapses double-tagged at P0 (grey bar) and P5 (blue bar). Grey data points are the number of synapses, and colored data points are averages from individual mice (*N* = 4 at P0 and *N* = 3 at P5). Significance: **P < 0.01; Unpaired *t* test. **(D)** Single optical plane confocal images of immunoreactivity against Iba1 (blue), C1q (green), CD47 (red), and VGluT1 (white) at P0 and P5. Inserts, higher magnification of the area indicated by dotted box. Yellow arrows indicate VGlut1 residues within microglia tagged by C1q and CD47. Red arrows indicate VGluT1 residues within microglia tagged by CD47 only. Scale bar: 5 μm. **(E)** 3D reconstructions of microglia (Iba1; yellow) and CD68 (red), showing the presence of CD68 within microglia. **(F)** Percentage of microglia in the LMC L4 motor neuron region containing VGlut1 residues tagged with CD47. Each data point represents a mouse (P0 and P10: *N* = 3; P5: *N* = 4). ***P < 0.001, one-way ANOVA, Tukey’s post hoc test. **(G)** Percentage of CD68 volume within microglia. Grey data points represent the number of microglia analyzed. Colored data points are the average from individual mice (*N* = 3 mice/group). **(H)** Microglia volume in WT, C3^−/−^, and CD47^−/−^ mice. Grey points are the number of microglia analyzed, and colored data points are the average from individual mice (*N* = 3 mice/group). Significance: **P < 0.001, ***P < 0.001; ns: no significance; one-way ANOVA with multiple comparisons using Bonferroni’s test. **(I)** 3D reconstruction of microglia obtained from z-stack confocal images (inset) from C3^−/−^ and CD47^−/−^ mice. The 3D reconstruction depicts Iba1 (cyan), CD68 (yellow), and VGluT1 (red); inset shows immunostaining for Iba1 (green), CD68 (red), and VGluT1 (blue). Scale bars: 5 μm. **(J)** Percentage of microglia volume filled with either CD68 or VGluT1 in C3^−/−^ and CD47^−/−^ mice. ns: no significance. Unpaired *t* test.

Since we observed higher CD47 tagging in the homozygous C3^−/−^ or C1q^−/−^ mice, we next investigated whether CD47 and classical complement are mutually exclusive for synaptic elimination. To test this, we performed immunohistochemistry for C1q and CD47 in the spinal cord of neonatal (P0–P5) mice. Remarkably, we found that both CD47 and C1q can tag the same VGluT1 synapse (yellow circles in Fig. 9, A and B). We also found synapses that were tagged either by CD47 or C1q alone (red circles for C1q and green circles for CD47 in Fig. 9, A and B). This type of synaptic tagging increased as animals aged from P0 to P5 (Fig. 9 C).

Since synapses tagged by C1q are susceptible to being engulfed by microglia (Stevens et al., 2007; Vukojicic et al., 2019), we next investigated the possibility that synapses tagged by C1q and CD47 could also be eliminated in a microglia-dependent manner. If CD47 played a protective role in synapse elimination, as reported in brain region (Lehrman et al., 2018), by extension, it should prevent engulfment by microglia. In such a case, it is expected that no CD47 should be found within microglia profiles. In contrast to this hypothesis, we found microglia containing VGluT1^+^ synaptic remnants tagged by both CD47 and C1q at both P0 and P5 (yellow arrows in Fig. 9 D). Remarkably, we also identified VGluT1^+^ residues associated with only CD47 inside microglia, especially at P5 (red arrows in Fig. 9 D). Quantification of CD47 tagging VGluT1^+^ remnants within microglia at P0, P5, and P10 revealed a higher percentage of microglia containing tagged synaptic remnants at P5 compared with P0 or P10, suggesting that P5 is the age in which microglia exhibit their highest activity in synaptic pruning mediated by CD47 (Fig. 9 F). These results suggest that microglia could potentially engulf synapses via CD47 tagging, in parallel to a mechanism requiring tagging by both CD47 and C1q. It is therefore likely that both mechanisms operate independently of each other.

**Figure S5.**
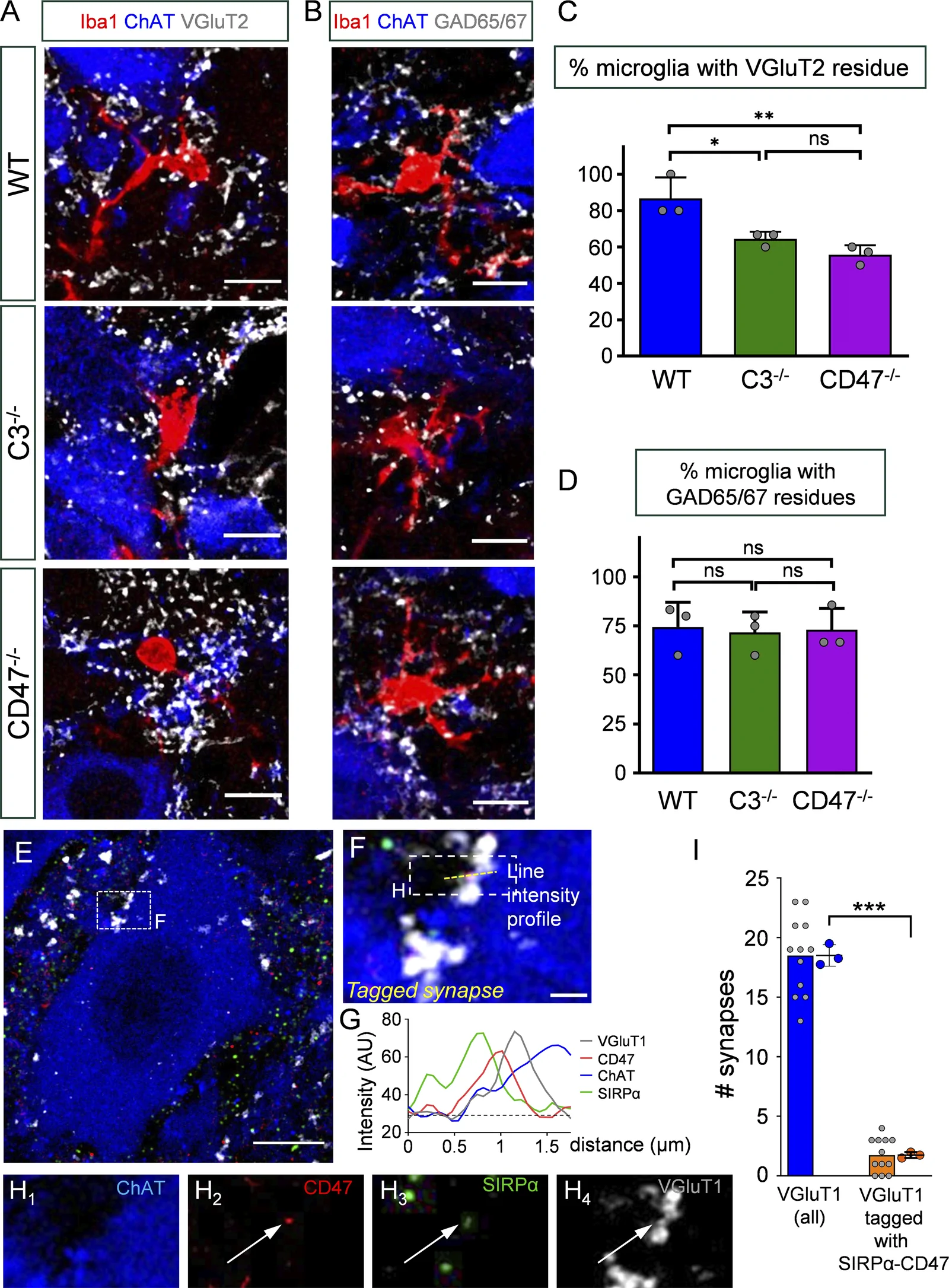
**Microglia engulfment of excitatory VGluT2^+^, but not inhibitory GAD65/67^+^ synapses, is affected by deletion of C3 or CD47, and SIRPα tags proprioceptive synapses on motor neurons. (A)** Confocal images of microglia (Iba1; in red) in the vicinity of L4 motor neurons (ChAT; in blue) and VGluT2^+^ synapses (in white) from WT (top), C3^−/−^ (middle), and CD47^−/−^ (bottom) mice. Scale bar: 10 μm. **(B)** Single-plane confocal images showing microglia (Iba1, in red), ChAT (blue) and GAD65/67^+^ (in white) synapses engulfed within microglia. Scale bar: 10 μm. **(C)** Percentage of microglia (in the vicinity of motor neurons) containing remnants of VGluT2^+^ synapses. WT, *N* = 3 mice; C3^−/−^, *N* = 3; CD47^−/−^, *N* = 3; *P < 0.05, **P < 0.01; one-way ANOVA, post hoc Bonferroni’s test. **(D)** Percentage of microglia containing GAD65/67 residues. WT, *N* = 3 mice; C3^−/−^, *N* = 3; CD47^−/−^, *N* = 3. Each data point corresponds to the average obtained from a single mouse. Only microglia within the motor neuron area was analyzed. ns: no significance. One-way ANOVA, post hoc Bonferroni’s test. **(E)** Confocal image showing quadruple immunoreactivity against CD47 (red), ChAT (blue), SIRPα (green), and VGluT1^+^ (white) in L4 WT motor neurons. Scale bar: 10 μm. **(F)** Inset from E, showing at higher magnification a VGluT1^+^ synapse contacting a motor neuron while being tagged by SIRPα and CD47. Scale bar: 1 μm. **(G)** Yellow dotted line indicates the line intensity profile shown in the graph (G) for all four fluorochromes confirming tagging of VGluT1 synapse by CD47 and SIRPα (overlap of green, red, and white lines). **(H**_**1**_**–H**_**4**_**)** Individual images from inset are shown in F. White arrows indicate point of tagging. **(I)** Grey data points in blue bar are the total number of VGluT1 synapses per motor neuron. Colored data points are the average per mouse. Grey data points in orange bar are the number of VGluT1^+^ synapses tagged by both CD47 and SIRPa for the same motor neurons analyzed (WT mice at P5). Colored data points represent the average number of synapses per animal (*N* = 3). ***P < 0.001, unpaired *t* test.

As expected, remnants of VGluT2 and GAD65/67 synapses were also found within microglia (Fig. S5, A and B) but the percentage of microglia containing VGluT2-remnants were only significantly reduced in C3^−/−^ and CD47^−/−^ mice (Fig. S5 C) but not for GAD65/67-remnants (Fig. S5 D) implying that inhibitory synapses are refined by a different mechanism compared with VGluT1^+^ or VGluT2^+^ synapses.

Importantly, we identified interaction sites between SIRPα, CD47, and proprioceptive (PV^+^) synapses within the motor neuron pool in postnatal spinal cords (Fig. S5, E–I). We use line intensity optical profiling as the method to identify and subsequently quantify synapses that are tagged by CD47 and SIRPα. According to these stringent criteria, we found ∼10% of proprioceptive synapses are tagged by CD47 and SIRPα in WT mice at P5 (Fig. S5 I). This finding strongly suggests that a novel mechanism of synaptic engulfment by microglia is mediated by CD47 and its receptor SIRPα.

To provide further support for the involvement of microglia in synaptic elimination mediated by CD47, we investigated the protein expression of CD68, a protein previously reported to correlate with engulfment activity (Schafer et al., 2012). Using immunohistochemistry against Iba1 and CD68, confocal microscopy, and Neurolucida, we measured the volume of CD68 within microglia in WT, C3^−/−^, and CD47^−/−^ mice (Fig. 9 E). We found a significant reduction in the percentage of CD68 presence in microglia in C3^−/−^ and CD47^−/−^ mice compared with WT counterparts (Fig. 9 G), suggesting less synaptic engulfment in either C3 or CD47 knockout mice. In addition, there was no difference in the volume of microglia analyzed in any of the experimental groups (Fig. 9 H), indicating that activation of microglia was not altered across the three different groups. To delve deeper into the CD68 assessment and provide further evidence about the degradation stage and the involvement of microglia, we quantified the volume of CD68 and expressed it as percentage of the entire volume of microglia in C3^−/−^ and CD47^−/−^ mice (Fig. 9, I and J). Additionally, we also expressed the volume of VGluT1^+^ remnants as a percentage of the entire volume of microglia (Fig. 9, I and J). These data suggest that CD68 is not only associated with VGluT1^+^ synapses but may also be associated with other types of synapses being removed, for example VGluT2^+^, as we show in Fig. S5 C.

**Figure 10.**
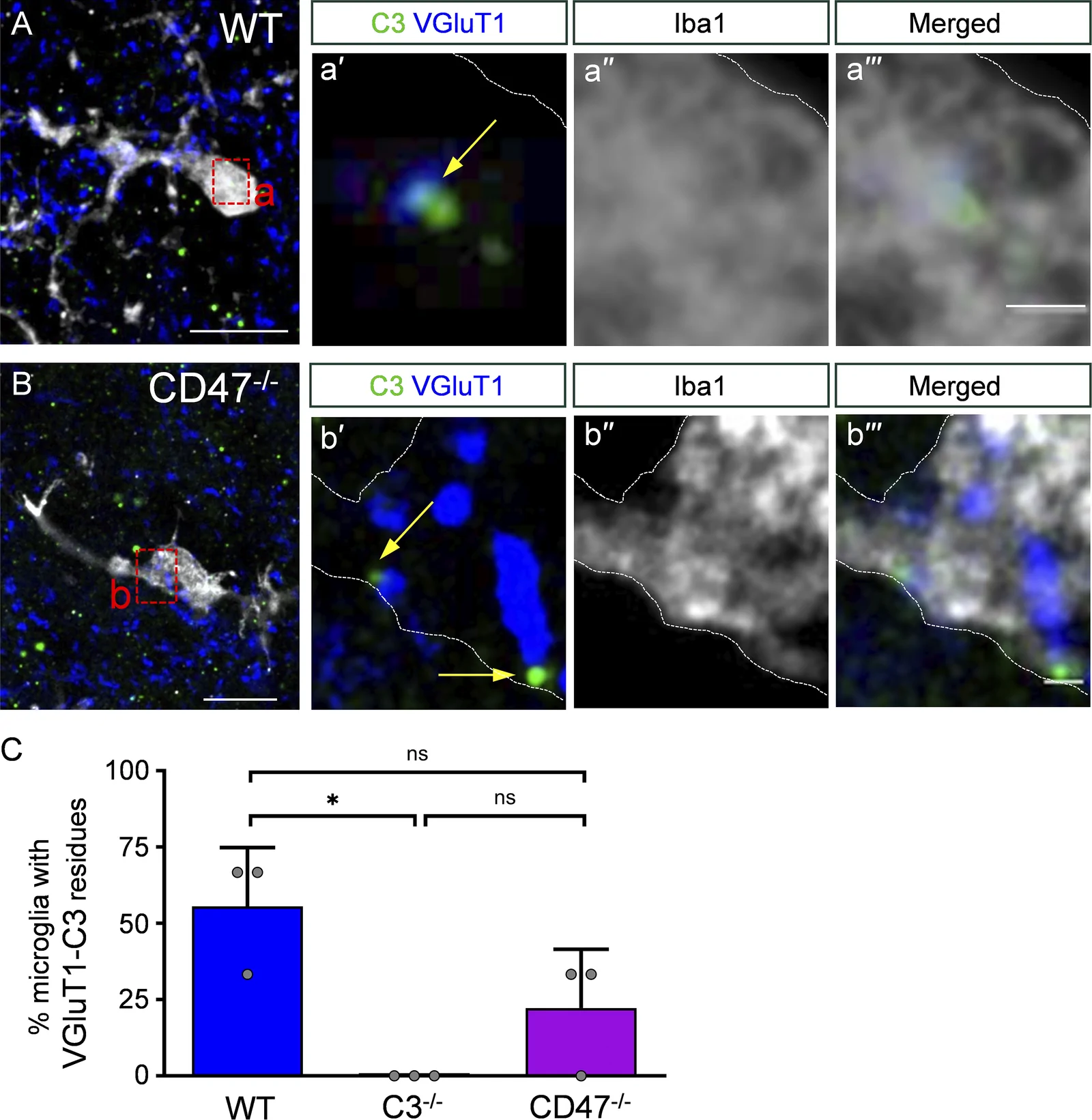
**Microglia engulfment of VGluT1 synapses tagged by C3. (A and B)** Single optical plane confocal images showing immunostaining against VGluT1^+^ (blue), Iba1 (white), and C3 (green) in WT (A) and CD47^−/−^ (B) mice (scale bar for A and B: 10 μm). Insets a and b (red rectangles) depict higher magnification images within microglia. a′ and b′: show C3 (green) and VGluT1 (blue), a″ and b″: Iba1 (white), and a‴ and b‴: show the merged image. Yellow arrows indicate tagged VGluT1-C3 residues. Scale bar for insets: 1 μm. **(C)** Quantification of percentage of microglia containing VGluT1-C3 residues. Analysis performed from sampled microglia within the motor neuron area. WT, *N* = 3 mice; CD47^−/−^, *N* = 3 mice. *P < 0.05; ns: no significance; one-way ANOVA with multiple comparisons using Bonferroni’s test.

We next assessed whether C3-tagged VGluT1 remnants could be found within microglia. We performed immunoreactivity experiments against C3, VGluT1, and Iba (Fig. 10, A and B). Quantification of the percentage of microglia containing C3-tagged VGluT1^+^ synapses in WT, CD47^−/−^, and C3^−/−^ (as controls) mice revealed a similar percentage of microglia containing C3-tagged synapses between WT and CD47^−/−^ mice (Fig. 10 C).

Taken together, these results demonstrate that complement cascade is not the only source of synaptic elimination during normal development, but importantly, we implicate a previously unknown and unforeseen role for CD47, which, instead of synapse protection, contributes to synaptic pruning. Furthermore, the classical complement and CD47-mediated molecular mechanisms are not mutually exclusive and could operate in a cooperative manner. This liaison ensures refinement of sensory-motor circuits during early spinal cord development presented by a dual fail–safe mechanism.

## Discussion

The presence and role of supernumerary synapses during spinal cord development have not been well understood. Here, we demonstrate that during early postnatal development, an excessive number of proprioceptive sensory synapses impinge on spinal motor neurons. These extra synapses make inappropriate contacts with motor neurons, resulting in miswired immature sensory-motor circuits. The emergence of mature sensory-motor circuits is regulated by two molecularly distinct but cooperating mechanisms. The first employs activation of the classical complement cascade, through the C1q–C3 pathway, while the second utilizes CD47-SIRPα signaling. The involvement of CD47 in synaptic elimination within the spinal cord is an unexpected function and in complete contradiction to the don’t eat me or protective function in brain regions. Taken together, our study proposes that the natural course of elimination of inappropriately generated synapses during early spinal cord development in mice employs a dual fail–safe system to ensure the emergence of mature spinal reflexes and normal motor control.

### Immature spinal sensory-motor circuits are miswired during early development

We report that genetic removal of the classical complement proteins, C1q and C3, as well as the integrin-associated protein CD47, results in ∼30% higher incidence of sensory synapses within the motor neuron pools in the developing lumbar spinal cord compared with normal mice. Our observations demonstrate that supernumerary synapses are established during embryonic development and subsequently pruned during postnatal development. The origin of these supernumerary synapses is proprioceptive, as well as, some excitatory (VGluT2^+^) synapses of unknown origin. Despite previous reports of transient inappropriate sensory synapses (Seebach and Ziskind-Conhaim 1994; Mendelsohn et al., 2015; Vukojicic et al., 2019; Gibson and Clowry 1999), their precise origin and role are not well defined. Here, we establish that proprioceptive sensory neurons make inappropriate synaptic contacts with motor neurons innervating a biomechanically antagonistic muscle. Supernumerary synapses appear to be predominant inappropriate, since the amplitude of the H-reflex in the homonymous muscle between WT, C3^−/−^, and CD47^−/−^ homozygous mice was similar. In addition, the origin of inappropriate synapses could be from several antagonistic muscles that are biomechanically similar in function (i.e., TA and extensor digitorum longus muscles) (Mendelsohn et al., 2015).

It has been previously reported that miswired neuronal circuits may be the result of mislocated postsynaptic sites. In the case of a spinal sensory-motor circuit, the *FoxP1* gene can control the spatial and settling position of motor neurons, leading to nonspecific sensory-motor circuits (Sürmeli et al., 2011). However, our analysis in the TA and Gs motor neuron pools did not reveal any statistically significant differences in the final settling position of their motor neuron somata when C3 or CD47 were genetically eliminated ubiquitously in mice. Importantly, we show that the inappropriate synapses are functional and exert an influence on the intrinsic firing properties of motor neurons they impinge upon. This miswired and immature circuit results in improper muscle contraction and negatively impacts the initial development of righting behavior. Although the inappropriate synapses still persist in both C3^−/−^ and CD47^−/−^ homozygous mice beyond the first postnatal week, the righting behavior is normalized because this behavior depends on the development of the vestibulospinal pathways, which mature and become dominant after the first postnatal week (Fletcher et al., 2017). The impact of inappropriate synapses and miswired circuits during adulthood remains to be elucidated. Nevertheless, we showed that the pruning of inappropriate synapses is an essential constraint for the emergence of mature sensory-motor circuits and normal motor behavior.

### Complement protein C3 mediates synapse elimination in sensory-motor circuits

Our study uncovers two molecularly distinct mechanisms that are causally responsible for the elimination of inappropriate synapses. One of these involves activation of the classical complement cascade through C1q–C3 signaling. The function of C3 in synaptic pruning has also been reported to be involved in the retinogeniculate system (Schafer and Stevens 2013). However, does the involvement of C3 protein result in indiscriminate pruning of synapses? The answer appears to be negative, since in the striatum in Huntington's disease, C3 has been implicated in the elimination of VGluT1^+^ synapses, leaving VGluT2^+^ synapses unaffected (Wilton et al., 2023). In our study, we demonstrated that VGluT1^+^ synapses are eliminated, suggesting that C3 is not responsible for the removal of selective synapses. However, it is possible that specific types of proprioceptive synapses, such as Ia, Ib, or II (de Nooij and Zampieri 2023), may be selectively targeted for elimination by C3. This observation raises the possibility that the selection of synapses to be eliminated is made by the upstream protein in the classical complement cascade, C1q. Alternatively, C3 may play a different role in synaptic elimination depending on regional differences between the brain and spinal cord.

Although C3 is a downstream target of C1q, it can also be produced by the lectin and alternate pathways (Reid 1985; Dodds 2002). However, it is unlikely that the latter pathways can be involved in synaptic pruning since they are associated with infection. A previous study in the brain demonstrated that synapse elimination is mediated by C3 activation and interaction with CD11b (C3 receptor) from microglia (Bollinger and Wohleb 2019). In our study, C3 is involved in the elimination of inappropriate synapses similar to C1q. Intriguingly, C3-mediated synapse elimination appears to be focused on somatic synapses rather than dendritic ones. This observation may offer an insight into the way the classical complement targets synapses for elimination according to their postsynaptic site.

An important question regarding the role of inappropriate synapses relates to their function. Do inappropriate synapses transmit, and if so, are they similar to the appropriate ones? Our functional assays argue that inappropriate synapses function to a similar level as the appropriate synapses. This observation indicates that C1q or C3 are not merely tagging dysfunctional or silent synapses as part of the clearance process. Instead, it suggests that inappropriate synapses might express a signal, sensed by the classical complement proteins, that is not present or detectable in the appropriate synapses.

### CD47 causes synapse elimination in the spinal cord, unlike its neuroprotective function in the brain

During the last decade, CD47 and its receptor SIRPα have been described as a don’t eat me signal in the central nervous system, in particular, in areas such as the hippocampus, retinogeniculate circuit, and cortex (Lehrman et al., 2018; Li et al., 2021; Shui et al., 2022). In striking contrast, however, our study demonstrates a novel and unexpected function for CD47 within the spinal cord. Should CD47 act as a neuroprotective agent in the spinal cord, its ablation would have increased synapse elimination as reported in brain regions. Remarkably, CD47^−/−^ animals exhibited the opposite effects, with its depletion inducing the same level of synaptic pruning as genetic deletion of the classical complement proteins C3 and C1q.

A common assumption regarding the protective function of CD47 implicates SIRPα. CD47 is expressed in proprioceptive neurons, motor neurons and spinal neurons, but not in glia (astrocytes or microglia). SIRPα however, is expressed by microglia and contacts CD47, implying a similar—if not identical—mechanism of CD47 interaction. In addition, SIRPα is present in proprioceptors, suggesting that the synapses on motor neurons destined to be pruned might express SIRPα. However, how inappropriate synapses that contact antagonistic motor neurons are selectively pruned through CD47-SIRPα signaling remains to be elucidated and future experiments knocking out from select cellular types might provide insights to this question. It is tempting to speculate regarding the possibilities of how CD47 could exert an engulfment-promoting function. Firstly, since previous studies in the brain did not investigate the classical complement proteins at the time when CD47 was investigated, it is possible that compensatory interactions of these complement proteins may affect CD47 activity. Second, some studies are contradictory regarding the function of CD47. For example, Lehrman and colleagues demonstrated that CD47 reduces VGluT2^+^ synapses but had no effect on VGluT1^+^ synapses in dLGN (Lehrman et al., 2018). In contrast, Ding and colleagues reported that elimination of CD47 affected VGluT1 synapses in the hippocampus (Ding et al., 2021). These reports raise the possibility that the effect of CD47 is not identical in different parts of the brain. Thus, it is possible that CD47 may play a different role in the spinal cord (as we show here) compared with different brain regions. Third, our study is focused on the effect of CD47 with regards to inappropriate synapses during the peak of the synaptic pruning period. It is possible that the sensory-motor circuit we investigated in our study to be different in the refinement period to those neuronal circuits in either dLGN, visual cortex, or hippocampus. Lastly, although previous studies reported on the activity-dependent effect of CD47 (Lehrman et al., 2018; Ding et al., 2021), none of these studies demonstrated the engulfment effect of microglia, raising the possibility that in the spinal cord, it may not be a difference between “weak” and “more active” synapses as in the brain.

Nevertheless, our data are consistent with the interpretation that CD47 is a major player in synaptic elimination of inappropriate synapses in the developing spinal cord. Although CD47 acts similarly to the classical complement proteins in eliminating inappropriate synapses, a distinct difference between the two mechanisms may be the postsynaptic site—soma versus dendrite—in which synapses are eliminated. To this end, CD47 tends to remove somatic synapses, whereas C3 appears to be mostly involved in the pruning of dendritic synapses. However, our results demonstrate that both participate in synaptic elimination regardless of whether it is somatic or dendritic. The significance of our study is underlined by the unanticipated function of CD47 in synapse elimination in spinal neuronal circuits, which suggests that the classical complement cascade acts alongside with CD47, providing a steadfast fail–safe dual mechanism in the elimination of inappropriate synapses.

### Limitations of the study

One limitation of our study is the uncertainty of the precise number of proprioceptive synapses on motor neurons originating from biomechanically synergistic muscles (i.e., EDL muscle to TA muscle) since we only studied either homonymous (i.e., TA muscle) or biomechanically antagonistic muscles (i.e., Gs muscle to TA muscle). We also did not investigate the effect of selective genetic elimination of C3 or CD47 from either glia or neurons to provide insights for the relative contribution of these two cellular types in synaptic elimination. Lastly, although our study provides strong evidence regarding the unexpected role of CD47 in aiding synaptic elimination in the spinal cord, it does not address why CD47 has a protective role for synapses in brain regions.

## Materials and methods

### Mice

Mice were housed under pathogen-free conditions, and all surgical procedures were performed on postnatal mice in accordance with the National Institutes of Health (NIH) Guidelines on the Care and Use of Animals and approved by the Columbia Animal Care and Use Committee (IACUC). Animals of both sexes were used in this study. CD47^−/−^ knockout mice were obtained from Jackson Laboratory (B6.129s7-Cd47^tm1Fpl^/J; Strain #003173). C3^−/−^ mice were obtained from The Jackson Laboratory (B6 129S4-C3^tmCrr^/J; Strain #003641). C1qa^−/−^ mice were provided by Dr. M. Botto (Imperial College London, London, UK) as previously reported (Botto et al., 1998) under an Material Transfer Agreement (MTA) agreement.

### Immunohistochemistry

Dissections of the spinal cords were performed on P7 animals. Mice were anesthetized using 1.2% Avertin (300 mg/kg) by intraperitoneal injection, and transcardial perfusions were performed with PBS followed by 4% paraformaldehyde (PFA). Postfixation with 4% PFA was performed overnight. The spinal cords were washed out with PBS and immersed in 5% agar, and transverse sections were collected from L4 to L5 spinal segments using a vibratome (Leica VT1000S) at 55 μm thickness. Sections were incubated in blocking serum containing 10% normal donkey serum in 0.01 M PBS with 0.3% Triton X-100 (PBS-T) for 1 h. Sections were incubated overnight at room temperature with primary antibodies at different concentrations in blocking serum. Sections were washed with PBS-T, and secondary antibodies were incubated in blocking serum for 3 h. Finally, sections were washed with PBS and mounted on glass slides using Fluoromount mounting medium (F4680; Sigma-Aldrich) and subsequently scanned on a aconfocal microscope.

For quadruple staining, an incubation with donkey anti-goat biotinylated antibody was performed after the incubation with primary antibodies in blocking serum for 3 h. After washing with PBS-Triton, Streptavidin–405 was added as secondary antibody following the protocol for a secondary antibodies described above. When anti-mouse antibodies were used, the tissue sections were incubated for 2 h with mouse-anti-mouse blocking serum (BMK-2202; Vector laboratories) at ∼50 μm/2.5 ml PBS.

The antibody concentrations used in this study are as follows: VGluT1 polyclonal anti-guinea pig (1:2,000; custom made) produced by Covance, designed against the epitope (C)GATHSTVQPPRPPPP, which lies within the N terminus of mouse VGluT1. The VGluT1 antibody was validated in VGluT1 knockout tissue (Fletcher et al., 2017). ChAT polyclonal anti-goat (1:200; AB144P; Millipore). CD47 monoclonal anti-rat (1:500; 555297; BD Pharmingen). C3 polyclonal anti-rabbit (1:500; PA5-21349; Thermo Fisher Scientific). GFP polyclonal anti-chicken (1:500; 1020; Aves Labs). Ds-red polyclonal anti-rabbit (1:200; 632496; Takara). C1q monoclonal anti-rabbit (1:1,000; AB182451; Abcam). Iba1 polyclonal ant-goat (1:500; ab5076; Abcam). Iba1 polyclonal anti-rabbit (1:500; 019-19741; Wako). GFAP polyclonal anti-rabbit (1:500; Z0334; Dako). Donkey anti-goat biotylinated (1:250; 705065147; Jackson Immuno Research Labs). VGluT2 monoclonal anti-mouse (1:250; MAB5504; Millipore). GAD65/67 monoclonal anti-mouse (1:1,000: ADI-MSA-225; Enzo). For secondary antibodies from Jackson Immuno Research Labs concentration used was 1:250: DyLight-405 Streptavidin (016-470-084). DyLight–488 donkey anti-chicken (703-545-155). DyLight–488 donkey anti-rabbit (711-545-152). Cy3 donkey anti-rat (712-165-153). Cy3 donkey anti-rabbit (711-165-152). Cy5 donkey anti-guinea pig (7066-175-148). Cy5 donkey anti-rabbit (711-175-152). Cy5 donkey anti-chicken (703-175-155).

### Labelling of muscle-selective motor neurons and proprioceptors

To label specifically motor neurons innervating the TA or Gs muscles and the proprioceptor-to-motor neuron synapses from antagonistic muscles, we used a modified protocol reported by Balaskas and colleagues (Balaskas et al., 2019). Briefly, two mouse strains were crossed: Pv-directed FlpO recombinase (Pv::FlpO), Jackson Laboratory (B6.Cg-*Pvalb*^*tm4.1(flpo)Hze*^/J) (ref. 022730), and Cre and Flp-dependent TdTomato reported mouse [Ai65 (RCFL-tdt)-D], Jackson Laboratory (B6;129S-*Gt(ROSA)26Sor*^*tm65.1(CAG-tdTomato)Hze*^/J) (ref. 021875), to obtain Pv::FlpO;Ai65 mice. These animals were crossed with C3^−/−^, C1q^−/−^, and CD47^−/−^ animals to obtain Pv::FlpO;Ai65;C1q^−/−^, Pv::FlpO;Ai65;C3^−/−^, and Pv::FlpO;Ai65;CD47^−/−^ strains, respectively. Only homozygous animals for all the genes were used for experiments, and Pv::FlpO;Ai65 was used as a control. Labelling of motor neurons innervating TA muscle and proprioceptors innervating Gs was performed as follows: Newborn (P0–P1) pups were anesthetized with isoflurane 5% and 1 l/min of O_2_. 3-mm incisions were performed unilaterally in the right hindlimb at the front and back sides of the hindlimb to expose the TA and Gs, respectively. ∼1 μl of the virus was injected using a fine glass micropipette. The viruses injected were (1) in TA AAV6-GFP (Vector core of the University of North Carolina at Chapel Hill, Chapel Hill, NC, USA) to label TA motor neurons; (2) CAV2-Cre virus in Gs (Plateforme de Vectorologie de Montpellier, Montpellier, France) to flip the expression of the Ai65 gene only in PV-positive Gs innervating cells; this allowed us to specifically label proprioceptors originating in a select muscle. To label proprioceptive synapses on motor neurons from homonymous muscles, we used the same protocol described above by injecting both, AAV6-GFP and CAV2-Cre viruses in the same TA muscle of Pv::FlpO;Ai65, Pv::FlpO;Ai65;C3^−/−^, and Pv::FlpO;Ai65;CD47^−/−^ strains. Animals were euthanized and samples were analyzed at P7–P8.

### Quantification of motor neurons, synapse number, and CD47 tagging

Images from immunohistochemistry experiments were obtained using an SP8 Leica confocal microscope (Leica Microsystems). Z-stack images of 20–30 μm at 3.5-µm interval, were obtained using a 40× oil immersion objective at 4,096 × 4,096 dpi resolution. Images were analyzed using ImageJ software (NIH). To quantify number of motor neurons, we used L4 sections from immunohistochemistry experiments at P5 from each mouse line. Quantification was performed unilaterally on ChAT-positive motor neurons on 75-μm thick sections. Z-stack images were obtained using a SP8 Leica confocal microscope (Leica Microsystems), with a 0.35 μm distance between pictures. Quantification of synapses number was performed using only neurons whose somata were completely covered by the Z-stack, and only synapses on the soma were quantified. For synapses on dendrites, the first 50-μm dendritic compartment from the soma was included in the analysis. Synapses were labelled as “tagged” by CD47 only or CD47 and C1q when their protein expression colocalized with the synaptic signal, identified by VGlut1, and confirmed by an overlap using intensity profiling. Analysis of the total number of synapses on somata and dendrites and the number of synapses tagged were used to obtain the percentage of tagged synapses.

### RNAscope assay

Spinal cords were obtained at P1, P5, and P10 pups, as was described for immunohistochemistry experiments. After overnight 4% post-fixation, the tissue was immersed in 10% sucrose in PBS at 4°C for 72 h. The tissue containing L4–L5 lumbar enlargement was obtained and frozen in Optimal Cutting Temperature media and sections of 14–20 μm were cut in a cryostat (Leica SM3050S) and mounted on SuperFrost Plus slides (Thermo Fisher Scientific). RNAscope Multiplex Fluorescent Detection Kit version 2 (Cat 323110; ACD Bio-Tchne) was used for detection. Slides were incubated at 60°C for 30 min and post-fixed with 4% PFA. Slides were dehydrated using ethanol dilutions of 50%, 70%, and 100% for 5 min each, followed by incubation with hydrogen peroxide (Ref. 322335; ACD Bio-Techne) for 10 min, and after washing out with distilled H_2_O, sections were embedded in 100% alcohol for 3 min. Protease Plus (Ref. 322331; ACD Bio-Techne) was added, and slides were incubated at 40°C for 30 min. Hybridization was performed by adding a specific probe for each gene and incubating the slides for 2 h at 40°C. Slides were washed with 1× wash buffer (Ref. 310091; ACD Bio-Techne) and incubated at room temperature overnight in 1× saline sodium citrate. The following day, slides were incubated with Hybridize AMP1, hybridize AMP2, and hybridize AMP3 for 30 min at 40°C each. Finally, HRP signals were developed using HRP-C1, HRP-C2, or HRP-C3 depending on the channel of each probe and incubated for 15 min at 40°C. TSA fluorescein, TSA Cy3, or TSA Cy5 (PerkinElmer. Ref. NEL760001KT) were used to reveal the probe following the company’s indications. To stop the hybridization, slides were incubated for 15 min at 40°C with an HRP blocker (Ref. 323107; ACD Bio-Techne) and mounted using Fluoromount mounting media (F4680; Sigma-Aldrich).

For double RNAscope–immunohistochemistry assays, the immunohistochemistry protocol was performed as indicated above, starting with the blocking using blocking serum after the incubation with HRP blocker. Images were taken between 1 and 15 days after the experiment.

Probes used were obtained from ACD Bio-Tchne and the protocol for dilution was followed as indicated by the company. Probes were C1q (Ref. 441221-C3; ACD Bio-Techne), CD47 (Ref. 515461-C2; ACD Bio-Techne), and SIRPα (Ref. 527228-C1; ACD Bio-Techne).

### CD68 expression in microglia and volumetric analysis

We performed immunohistochemistry experiments to study the expression of CD68 in microglia in WT, C3^−/−^, and CD47^−/−^ mice at P5. The immunohistochemistry protocol used was the same as described above. Primary antibodies and concentrations used were CD68 monoclonal antibody anti-rat (1:500, Bio-Rad, MCA1957), Iba1 polyclonal anti-rabbit (1:500, 019-19741; Wako), and ChAT polyclonal anti-goat (1:200, AB144P; Millipore). Secondary antibodies used were DyLight–488 donkey anti-rat (712-475-153), Cy3 donkey anti-rabbit (711-165-152), and Cy5 donkey anti-goat (705-175-147). All secondary antibodies were purchased from Jackson Immuno Research Labs. Images were obtained using an SP8 Leica confocal microscope (Leica Microsystems). Z-stack images for 15–20 μm at 0.35-µm interval were obtained using a 40× oil immersion objective at 4,096 × 4,096 dpi resolution. Further, the images were analyzed and processed using Neurolucida software, and 3D images were created to measure the volumetric distribution of microglia and CD68 expression inside microglia. Only microglia surrounding L4–L5 ChAT^+^ motor neurons were taken into consideration for analysis. A minimum of five microglia, were analyzed per animal and CD68 expression in microglia was normalized by the total volume per cell.

### Genotype

DNA extraction was done using tail tissue incubated at 55°C for 60 min in 0.05% Proteinase K (Ref. EO0491; Thermo Fisher Scientific), diluted in tail lysis buffer containing Tris 1 M, EDTA 0.5 M, NaCl 0.2 M, and SDS 0.07 M. Samples were diluted at 1:20 in H_2_O. Genotype protocols for PV::FlpO, Ai65 (RCFL-tdt), C3^−/−^, C1q^−/−^, and CD47^−/−^ animals were followed as described on Jackson laboratory website (https://www.jax.org/). For all animals, universal PCR was used as follows: 12.5 μl of GoTaq Hot Start Green Master Mix (Promega), 0.5 μl of each primer (25 μM; Sigma-Aldrich), and 4 μl of 1:20 diluted lysed tail DNA in a final volume of 25 μl using dH_2_O. For Pv::FlpO and Ai65 (RCFL-tdt)-D and CD47^−/−^ alleles, products were amplified using the following thermal cycling method: 94°C for 2 min, followed by 10 cycles of 94°C for 20 s, 65°C for 15 s, 68°C for 10 s, then 28 cycles of 94°C for 15 s, 60°C for 15 s, 72°C for 10 s, and followed by 72°C for 2 min. For C3^−/−^ allele, products were amplified using the following thermal cycling method: 94°C for 3 min, followed by 12 cycles of 94°C for 20 s, 64°C for 30 s, 72°C for 35 s, then 25 cycles of 94°C for 20 s, 58°C for 30 s, 72°C for 35 s, and followed by 72°C for 2 min. For C1q^−/−^ allele, products were amplified using the following thermal cycling method: 94°C for 4 min, followed by 35 cycles of 94°C for 1 min, 57°C for 30 s, 72°C for 30 s, and followed by 72°C for 10 min.

Customized primers used are listed as follows: for C1q, mutant 5′-GGG​GAT​CGG​CAA​TAA​AAA​GAC-3′, WT 5′-GGG​GCC​TGT​GAT​CCA​GAC​AG-3′, and common 5-′ACC​AAT​CGC​TTC​TCA​GGA​CC-3′, with a product of WT ∼330 and mutant ∼120. For C3, mutant 5′-TGG​GCT​CTA​TGG​CTT​CTG​AG-3′, WT 5′-CAC​CTT​ACA​GCA​CTC​CCA​CA-3′, and common 5-′GAA​GTG​GAA​GTT​GAA​CAA​ATC​G-3′, with a product of WT ∼300 and mutant ∼199. For Ai65 (RCFL-tdt)-D, in separate reactions, WT 5′ACG​GGC​AGT​AGG​GCT​GAG-3′, 5′-AGC​CTG​CCC​AGA​AGA​CTC​C-3′, and mutant 5′GCA​ATA​GCA​TCA​CAA​ATT​TCA​C-3′, 5′-TCT​AGC​TTG​GGC​TGC​AGG​T-3′. For PV::FlpO, WT 5′GGA​TGC​TTG​CCG​AAG​ATA​AG-3′, mutant 5′CTG​AGC​AGC​TAC​ATC​AAC​AGG-3′, and common 5′-TGT​TTC​TCC​AGC​ATT​TCC​AG-3′. For CD47, WT 5′CAC​CTT​ACA​GCA​CTC​CCA​CA-3′, mutant 5′-TGG​GCT​CTA​TGG​CTT​CTG​AG-3′, and common 5′-GAA​GTG​GAA​GTT​GAA​CAA​ATC​G-3′.

### Electrophysiology

The ex vivo spinal cord preparation used in this study has been described in previous publications (Fletcher et al., 2017; Mentis et al., 2011). Briefly, neonatal pups were decapitated, and the spinal cord was extracted in artificial cerebrospinal fluid (aCSF) at ∼12°C and oxygenated with 95% O_2_/5% CO_2_. aCSF contains 128.35 NaCl, 4 KCl, 0.58 NaH_2_PO_4_.H_2_O, 21 NaHCO_3_, 30 D-glucose, 1.5 CaCl_2_.H_2_O, and 1 MgSO_4_.7H_2_O. The spinal cord was transferred to the recording chamber with aCSF perfusion (∼10 ml/min) at room temperature (∼22°C). Suction electrodes were placed in the dorsal root or ventral root for either stimulation or recording.

The stimulus threshold was defined as the minimal current necessary to evoke a response in the ventral root in three out of five trials, with 0.2 ms of stimulation duration. The stimulus was delivered by a constant current stimulus isolator (A365; WPI). The recordings were amplified 1,000× (CyberAmp, Molecular Devices), fed to an A/D interface (Digidata 1320A; Molecular Devices), and acquired at 50 kHZ using Clampex (version 10, Molecular Devices). Data were then analyzed offline using Clampfit (Molecular Devices). Only monosynaptic responses over 1 mV amplitude were considered for analysis. A series of 10 stimuli were performed at 0.1, 0.2, 1, and 10 Hz. Monosynaptic amplitude was measured as the average of 10 single responses at 0.1 Hz.

The ex vivo spinal cord–hindlimb preparation was prepared following the same protocol for the ex vivo spinal cord preparation. A marked difference was the careful dissection of the hindlimb in continuity with the sciatic nerve as well as the L4 and L5 dorsal and ventral roots. The sural nerve (pure sensory) was cut, and the CP and Tb nerves were intact. Concentric needle electrodes were placed within the TA and Gs muscles. Suction electrodes were positioned “en passant” on CP and Tb nerves. Ten stimuli were evoked at 0.1, 0.2, and 1 Hz frequencies. The amplitude was measured from baseline to pick, and its latency was measured from the onset of the stimulus artifact to onset of the response, which became higher than 3× SD of the baseline noise.

Whole-cell patch-clamp recordings were performed using ex vivo spinal cord–hindlimb preparation. To allow pure synaptic potentials (excitatory postsynaptic potentials) to be recorded and not be contaminated by antidromic action potentials from the CP nerve (for TA motor neuron recordings), the L4 and L5 ventral roots were cut and placed in suction electrodes. The spinal cord was placed on the lateral side to access motor neurons from the lateral funiculus. The dura mater was removed between the L4 and L5 areas. The intracellular electrode was guided by a motorized micromanipulator, guided by fluorescence signal from motor neurons retrogradely labelled at an earlier age and visualized with the aid of a Leica epifluorescent microscope (DM 6000FS). The intracellular solution contained (in mM): 130 K-gluconate, 10 NaCl, 10 HEPES, 1 EGTA, 1 MgCl_2_, 0.1 CaCl_2_, and 1 Na-GTP. pH was adjusted to 7.2 with KOH. Osmolality was adjusted to 290–295 mOsmol. Additionally, Alexa555 (1 mg/ml) and Neurobiotin (1 mg/ml) were added to the intracellular solution as to visualize the recorded cell post hoc. Borosilicate electrodes were pulled using a puller P-1000 (Sutter) with resistance ranging between 13 and 18 MΩ. The junction potential was corrected and taken into account for subsequent analysis. The input resistance for each cell was obtained from the slope of a steady-state (linear) current-voltage plot in response to a series of hyperpolarizing currents of 50 pA each. To confirm the recorded neuron physiologically as a motor neuron, stimulation of the ventral root resulted in antidromically evoked action potentials. Signals were acquired using a Digidata 1440A (Molecular Devices) controller with pClamp 10.3 software. EPSC and I-V responses were analyzed off-line with the software Clampex 10.1 (Molecular Devices).

### Spatial distribution of motor neuron populations

To identify the spatial distribution of TA and Gs motor neurons, a modified protocol previously described by Bikoff and collaborators was followed (Bikoff et al., 2016). Briefly, samples were obtained from P5 animals injected with CTB488 in TA and CTB555 in Gs at P0. Slides were cut to 75 μm, and immunohistochemistry against ChAT was performed in order to confirm that labelled cells were motor neurons. Confocal images were obtained from areas only between L4 and L5 and images in which both TA and Gs motor neurons were located in the same space and were used for analysis. Z-stack maximal projections were obtained using Image software. Images were analyzed using Neurolucida software. and seven consecutive z-stack images were used (450 μm total). Using the “LABEL” function, TA and Gs motor neurons were manually localized. Additionally, the spinal cord contour was delimited, and the central canal was taken as reference point 0.0 (in the x and y axes). Cartesian coordinates for each motor neuron were determined and obtained with the reference to the central canal. Coordinates were exported as .xls files and plotted using the MATLAB extension “make contours.” Analysis of the density distribution was performed based on the centroid of the population. The centroid was defined as the mean x and y coordinates across cells. The mean of the cell population distribution in the ventro-dorsal and the mediolateral aspects was obtained for each animal, and values were averaged for each population.

### Statistical analysis

Statistical analysis was performed using GraphPad Software (PRISM version 6.04). Comparisons were performed using either a two-tailed unpaired *t* test or one-way ANOVA, using Tukey’s as a post hoc test when conditions of normality and homoscedasticity were met. If violated, Mann–Whitney–Wilcoxon and Kruskal–Wallis tests were used as nonparametric tests. Post hoc multiple comparison methods are indicated in the Results and figure legends when necessary. We presented our results in two ways: (1) data points (in grey) with bars show all neurons analyzed in each group, and (2) colored data points ± standard error of the mean (SEM) show the average value measured for each mouse. Statistical comparison was performed amongst the average values from mice to avoid issues of pseudoreplication in the cases that we have measured synapses from many motor neurons across different numbers of mice. To test for homoscedasticity (equality of variance), we performed the Brown–Forsythe test for values of individual motor neurons in each mouse. Statistical significance is provided (actual P value) in each figure legend and assigned in the graphs on the following criteria: *P ≤ 0.05, **P ≤ 0.01, and ***P ≤ 0.001. Results are expressed as means ± SEM.

### Online supplemental material

In the supplementary information, we present five supplementary figures and three supplemental videos. Fig. S1 shows the effects of genetic deletion of C3 alone, CD47 alone, or combined in the sensory-motor circuit. Fig. S2 shows the exclusion criterion for inappropriate synapses, which are formed between TA proprioceptors onto Gs motor neurons, density maps for TA and Gs motor neuron pools, and no change in appropriate synapses in C3^−/−^ and CD47^−/−^ mice. Fig. S3 shows the H-responses evoked by antagonistic nerve stimulation in C1q^−/−^ mice and intrinsic properties and morphological identification of TA motor neurons in WT, C3^−/−^, and CD47^−/−^ mice. Fig. S4 shows the effects of C1q genetic deletion on the sensory-motor circuit. Lastly, Fig. S5 shows the microglia engulfment of excitatory VGluT2^+^, but not inhibitory GAD65/67^+^ synapses, being affected by deletion of C3 or CD47, and SIRPα tags proprioceptive synapses on motor neurons. Video 1 shows the righting times in WT, C3, and CD47 knockout mice. Video 2 shows the righting times in WT, C3 hets, CD47 hets, and C3+CD47 het mice. Video 3 shows the position of TA and Gs motor neurons within the ventral horn of spinal cord from analyzed spinal cord sections.

## Inclusion and ethics

One of the authors of this paper self-identifies as an underrepresented ethnic minority in science.

## Data Availability

All data are available from the corresponding author upon reasonable request.
